# Periarteriolar niches become inflamed in aging bone marrow, remodeling the stromal microenvironment and depleting lymphoid progenitors

**DOI:** 10.1073/pnas.2412317122

**Published:** 2025-03-10

**Authors:** Liming Du, Maria Angelica Freitas-Cortez, Jingzhu Zhang, Yuanyuan Xue, Reshma T. Veettil, Zhiyu Zhao, Sean J. Morrison

**Affiliations:** ^a^Children’s Research Institute and the Department of Pediatrics, University of Texas Southwestern Medical Center, Dallas, TX 75390; ^b^HHMI, University of Texas Southwestern Medical Center, Dallas, TX 75390

**Keywords:** hematopoietic stem cell, lymphoid progenitor, inflammation, interferon, niche

## Abstract

In early postnatal and young adult bone marrow, Leptin receptor–expressing (LepR^+^) stromal cells and endothelial cells synthesize factors required for hematopoietic stem cell (HSC) maintenance, including Stem Cell Factor (SCF) and Cxcl12. However, little is known about how these stromal cells change during aging. We performed single-cell RNA sequencing of mouse bone marrow stromal cells at 2, 12, and 24 mo of age. We identified five transcriptionally distinct subsets of LepR^+^ cells, all of which expressed the highest levels of *Scf* and *Cxcl12* in bone marrow throughout adult life. In aging bone marrow, SCF from LepR^+^ cells, but not endothelial cells, continued to be necessary for the maintenance of HSCs and early restricted progenitors. However, arteriolar endothelial cells and other periarteriolar cells expressed increasing levels of interferon during aging. This increased the numbers of periarteriolar *Sca1^+^Cxcl9^+^LepR^+^* cells with an inflammatory gene signature and depleted lymphoid progenitors, at least some of which are also periarteriolar. The periarteriolar environment thus became particularly inflamed during aging, remodeling the stromal microenvironment and depleting lymphoid progenitors in an interferon-dependent manner.

## Significance

Periarteriolar *Sca1^+^Cxcl9^+^LepR^+^* cells represent a subset of Leptin receptor–expressing (LepR^+^) cells that increased in number with age and were marked by interferon-regulated gene expression. Treatment of old mice with antibodies that blocked type I and type II interferon signaling depleted *Sca1^+^Cxcl9^+^LepR^+^* cells and increased the numbers of lymphoid progenitors. Increasing interferon expression thus contributes to the changes in bone marrow hematopoiesis during aging, remodeling the stroma and depleting lymphoid progenitors. These observations raise the possibility that inflammation is not uniform across aging bone marrow: It may preferentially affect certain microenvironments, including around arterioles, which serve as a niche for at least some lymphoid progenitors.

In early postnatal and young adult mice, Leptin receptor–expressing (LepR^+^) mesenchymal stromal cells and endothelial cells maintain HSCs and various restricted hematopoietic progenitors within perisinusoidal niches (1–6) as well as lymphoid progenitors within periarteriolar niches (7, 8) by producing Stem Cell Factor (SCF) (7, 9), Cxcl12 (10–12), pleiotrophin (13), and interleukin 7 (IL7) (14). These LepR^+^ cells have also been described as Cxcl12-abundant reticular (CAR) cells (15, 16). LepR^+^ cells promote vascular regeneration by producing Angiopoietin-1 (17) and VEGF-C (18) and the maintenance of peripheral nerves in bone marrow by producing nerve growth factor (19). Although HSCs require SCF produced by both LepR^+^ stromal cells and endothelial cells in young adult mice (2, 5, 9), the maintenance of many restricted hematopoietic progenitors depends only on SCF produced by LepR^+^ cells, not endothelial cells (5). Perisinusoidal niches for HSCs are preserved during aging (20), but the identity of the cells that produce SCF for HSC maintenance has not been functionally tested during aging and we have limited insight into how stromal cells change their properties during aging.

LepR^+^ cells arise perinatally from perichondral cells that participate in the formation of bone (21–23) and then expand in number throughout the bone marrow by early adulthood (2, 24). Although the frequency of all LepR^+^ cells in the bone marrow remains similar throughout adulthood, the frequency of Osteolectin^+^LepR^+^ cells, which create a periarteriolar niche for early lymphoid progenitors, declines during aging (7). RNA sequencing showed that *Scf* expression by LepR^+^ cells and endothelial cells does not change much during aging (25, 26), raising the question of what mediates the changes in hematopoiesis during aging.

One key driver of changes in hematopoiesis during aging is increased inflammation in the bone marrow (25–27). This includes increased production of multiple proinflammatory cytokines, including CCL5 (28, 29), IL1β (25), IL6 (27, 30), and TNF (30–34). These increases in proinflammatory cytokines contribute to the increase in myelopoiesis in aging bone marrow (25, 27, 28, 31). Some of these factors are produced by immune cells (35, 36) while others are produced by stromal cells (36, 37), though the location of the cells producing these factors in the bone marrow is unknown. Interferon expression also increases with age in many tissues (38, 39) and interferons are capable of promoting chronic HSC activation and depletion (34, 40–42). Indeed, the resistance of *Dnmt3a* mutant HSCs to the effects of inflammation contributes to their competitive advantage over wild-type HSCs after pathogen infection (43) or in the context of autoimmune disease (44). Nonetheless, to our knowledge, the effect of interferon on stromal cells and hematopoiesis in aging bone marrow has not yet been tested.

## Results

### The Stromal Cell Composition of the Bone Marrow Changes during Aging.

**Fig. 1.**
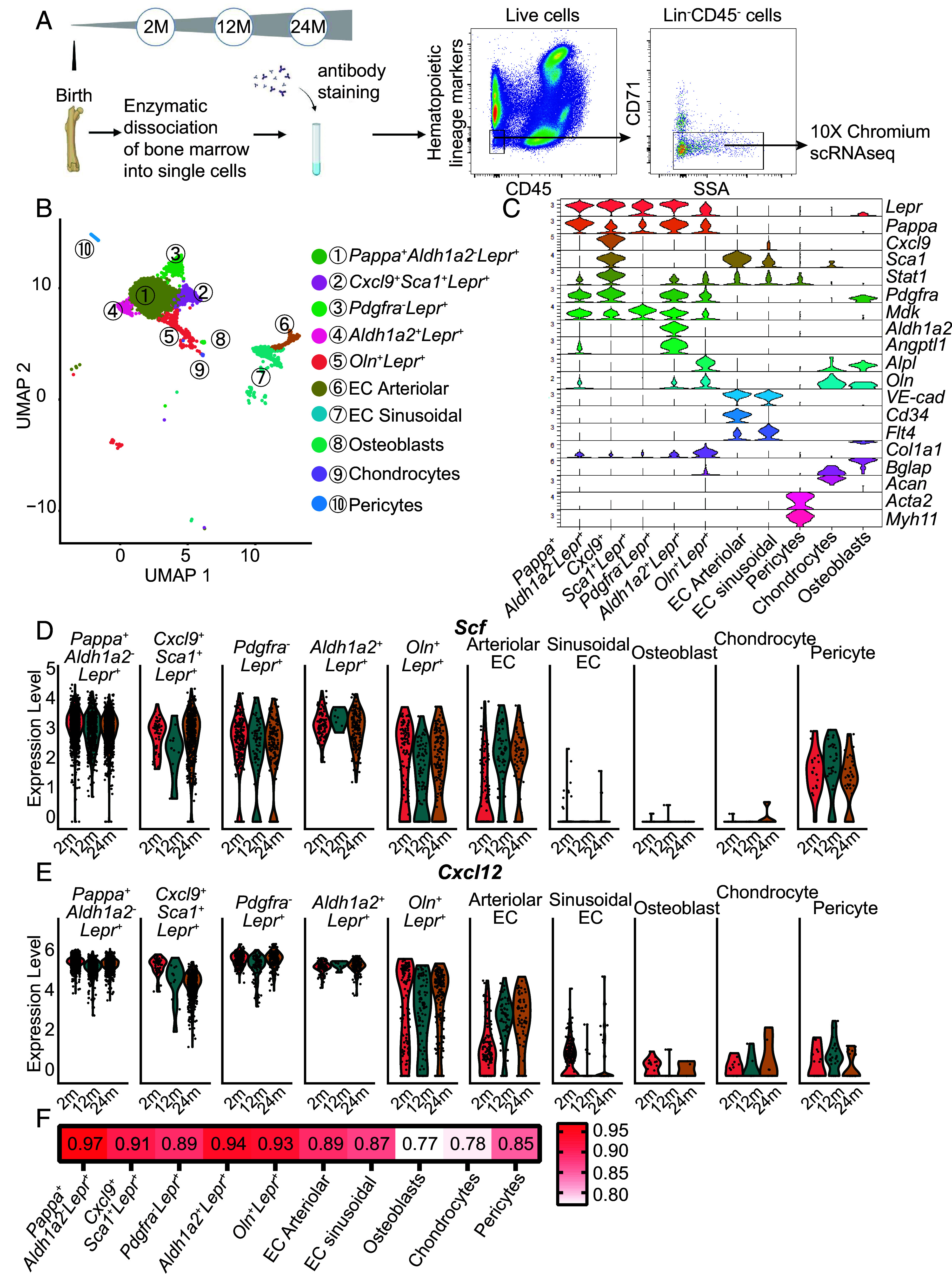
Single-cell RNA sequencing of stromal cells from the bone marrow of 2-, 12-, and 24-mo-old mice. (*A*) Experimental design and flow cytometry gates used to isolate bone marrow stromal cells that were negative for CD45, CD71, and hematopoietic lineage markers from enzymatically dissociated bone marrow from crushed femurs and tibias obtained from 2-, 12-, and 24-mo-old mice (three mice per age). Images were derived from BioRender. (*B*) Uniform manifold approximation and projection plot showing cell clusters from the analysis of a total of 5,507 bone marrow stromal cells from enzymatically dissociated 2-, 12-, and 24-mo-old mice bone marrow. (*C*) Violin plot showing the marker genes that distinguished each cell cluster. (*D* and *E*) Violin plots showing *Scf* (*D*) and *Cxcl12* (*E*) expression in each cell cluster at each age. (*F*) Spearman correlation coefficients comparing single-cell RNA sequencing data from 2- and 24-mo-old mice for each cell cluster.

To better understand the changes in bone marrow stromal cells during aging, we performed single-cell RNA sequencing on Lineage^−^CD45^−^CD71^−^ bone marrow stromal cells from 2-, 12-, and 24-mo-old mice (Fig. 1*A*). Femurs and tibias from mice of each age were crushed and enzymatically dissociated, then the Lineage^−^CD45^−^CD71^−^ stromal cells were isolated by flow cytometry. These cells represented approximately 0.6% of bone marrow cells at each age (*SI Appendix*, Fig. S1 *A–C*). After quality filtering of the sequence data, we included a total of 2,430 cells from three mice at 2 mo of age, 906 cells from three mice at 12 mo of age, and 2,171 cells from three mice at 24 mo of age in the analysis. We detected transcripts from a median of 2,218, 2,228, and 2,029 genes per cell at 2, 12, and 24 mo of age, respectively.

Unsupervised clustering identified 10 clusters of stromal cells (*SI Appendix*, Fig. S1*D*). Cell identity was assigned to each cluster based on the differential expression of known marker genes (2, 7, 8, 45–49) (Fig. 1*C* and *SI Appendix*, Fig. S1*E*). Five transcriptionally distinct clusters of LepR^+^ cells were identified (clusters 1 to 5 in Fig. 1*B*). In addition to the periarteriolar *Osteolectin^+^Lepr^+^* cells characterized in a prior study (7), we also found *Pappa^+^Aldh1a2^−^Lepr^+^* cells, *Cxcl9^+^Sca1^+^Lepr^+^* cells, *Pdgfra^−^Lepr^+^* cells, and *Aldh1a2^+^Lepr^+^* cells (Fig. 1 *B* and *C*). All of these *Lepr^+^* cell clusters strongly expressed *Scf* (Fig. 1*D*) and *Cxcl12* (Fig. 1*E*) at 2, 12, and 24 mo of age. Indeed, the average levels of *Scf* and *Cxcl12* in these *Lepr^+^* cell clusters were higher than any other bone marrow stromal cell population. The only other stromal cells that expressed appreciable levels of *Scf* and *Cxcl12* were arteriolar endothelial cells and periarteriolar pericytes, which expressed lower levels of *Scf* and *Cxcl12* than most *Lepr^+^* cells. All five subsets of *Lepr*^+^ cells also expressed *Foxc1* (*SI Appendix*, Fig. S2*A*) and *Ebf3* (*SI Appendix*, Fig. S2*B*), transcription factors necessary for the formation of LepR^+^/CAR cells and HSC niches (50, 51).

All cell clusters were transcriptionally similar across ages, with Spearman correlation coefficients between 0.77 and 0.97 (Fig. 1*F*); however, the correlation coefficients were higher in some populations than others. Among niche cell populations, sinusoidal endothelial cells, arteriolar endothelial cells, *Pdgfra^−^Lepr^+^* cells, and *Cxcl9^+^Sca1^+^Lepr^+^* cells exhibited more transcriptional changes during aging as compared to other *Lepr*^+^ cell clusters (Fig. 1*F*). Osteoblasts, chondrocytes, and pericytes exhibited the greatest transcriptional changes during aging (Fig. 1*F*).

To independently confirm that most *Scf* and *Cxcl12* were produced by LepR^+^ cells, irrespective of age, we aged *Scf*-GFP and *Cxcl12*-DsRed mice. The frequency and absolute number of LepR^+^ cells, identified based on anti-LepR antibody staining, did not significantly change in the bone marrow of mice at 2, 12, 18, and 24 mo of age (*SI Appendix*, Fig. S3 *A* and *B*). Approximately 95% of *Scf*-GFP^+^ cells were LepR^+^ and 84% of LepR^+^ cells were *Scf*-GFP^+^ at all ages (*SI Appendix*, Fig. S3 *C–J*). Approximately 97% of *Cxcl12*-DsRed^high^ cells were LepR^+^ and 71% of LepR^+^ cells were *Cxcl12*-DsRed^high^ at all ages (*SI Appendix*, Fig. S3 *K–R*). Thus, nearly all cells that expressed high levels of *Scf* or *Cxcl12* were LepR^+^ at all ages in adult bone marrow, consistent with the single-cell RNA sequencing. It is important to note, however, that adipocytes in adult bone marrow also express high levels of *Scf* (52). While adipocytes are rare in the long bones of young adult mice, they become more prevalent during aging (53). Since adipocytes are too large and fragile to detect by flow cytometry, it is possible that they contribute to the production of *Scf* in old adult bone marrow in a way that was not detected in these experiments.

To better understand the biological differences among these clusters of *Lepr^+^* stromal cells, we performed gene ontology (GO) term enrichment analysis. *SI Appendix*, Fig. S4 shows the eight most significantly enriched GO terms for each *Lepr^+^* cell cluster. *Pappa^+^Aldh1a2^–^Lepr^+^* cells expressed the highest average levels of *Scf* and *Cxcl12* among all cell clusters in the bone marrow (Fig. 1 *D* and *E*) and exhibited enrichment for GO terms related to hematopoiesis, suggesting that these cells create niche(s) for hematopoietic stem/progenitor cells (*SI Appendix*, Fig. S4*A*). In *Cxcl9^+^Sca1^+^Lepr^+^* cells, all eight of the most significantly enriched GO terms related to interferon-regulated genes, suggesting that these cells reside in an inflammatory microenvironment (*SI Appendix*, Fig. S4*B*). In *Pdgfra^–^Lepr^+^* cells, all eight of the most significantly enriched GO terms related to ribosome biogenesis, suggesting that these cells engage in higher rates of protein synthesis (*SI Appendix*, Fig. S4*C*), *Pdgfra^–^Lepr^+^* cells did not show an enrichment for genes related to cell division (*SI Appendix*, Fig. S5) and the frequency of these cells in the bone marrow did not change with age (*SI Appendix*, Table S1), suggesting that they were not rapidly dividing. In *Aldh1a2^+^Lepr^+^* cells, 4 of the top 8 GO terms related to extracellular matrix remodeling (*SI Appendix*, Fig. S4*D*). In *Osteolectin^+^Lepr^+^* cells, 4 of the top 8 GO terms related to osteogenesis, consistent with the observation that these cells are osteogenic progenitors (7) (*SI Appendix*, Fig. S4*E*).

### SCF from LepR^+^ Cells Is Required to Maintain HSCs During Aging.

**Fig. 2.**
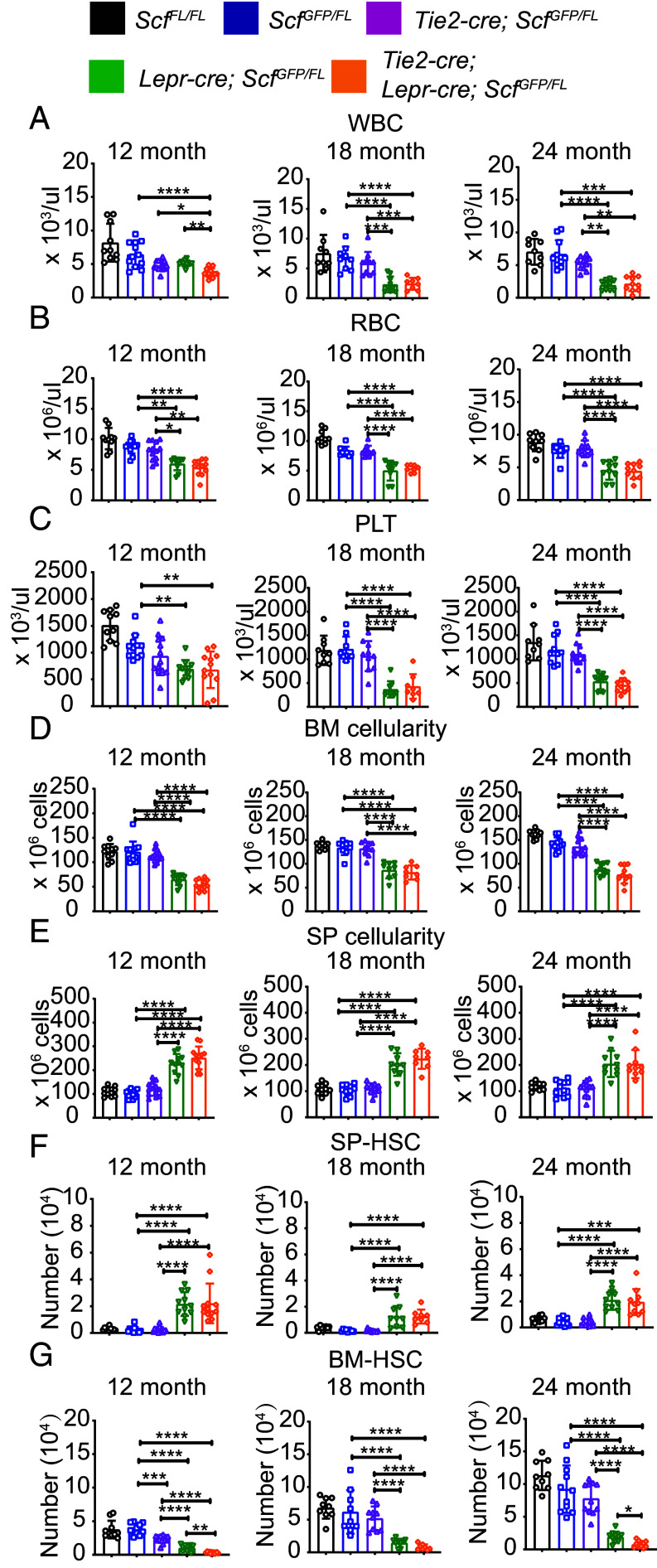
SCF from LepR^+^ stromal cells is required for the maintenance of HSCs and hematopoiesis in the bone marrow, but not the spleen, of 12-, 18-, and 24-mo-old mice. (*A–C*) WBC (*A*), RBC (*B*), and platelet (PLT) (*C*) counts in the blood of mice at 12, 18, and 24 mo of age. (*D*) Bone marrow cellularity (two tibias and femurs) of 12-, 18-, and 24-mo-old mice. (*E*) Spleen cellularity in 12-, 18-, and 24-mo-old mice. (*F*) Number of HSCs in the spleens of 12-, 18-, and 24-mo-old mice. (*G*) Number of HSCs in the bone marrow of 12-, 18-, and 24-mo-old mice. Each dot represents a different mouse. Each panel shows 10 to 13 mice per genotype in six independent experiments for 12-mo-old mice, 8 to 10 mice per genotype in five independent experiments for 18-mo-old mice, and 9 to 11 mice per genotype in six independent experiments for 24-mo-old mice. All data represent mean ± SD (*P < 0.05; **P < 0.01; ***P < 0.001; ****P < 0.0001).

**Fig. 3.**
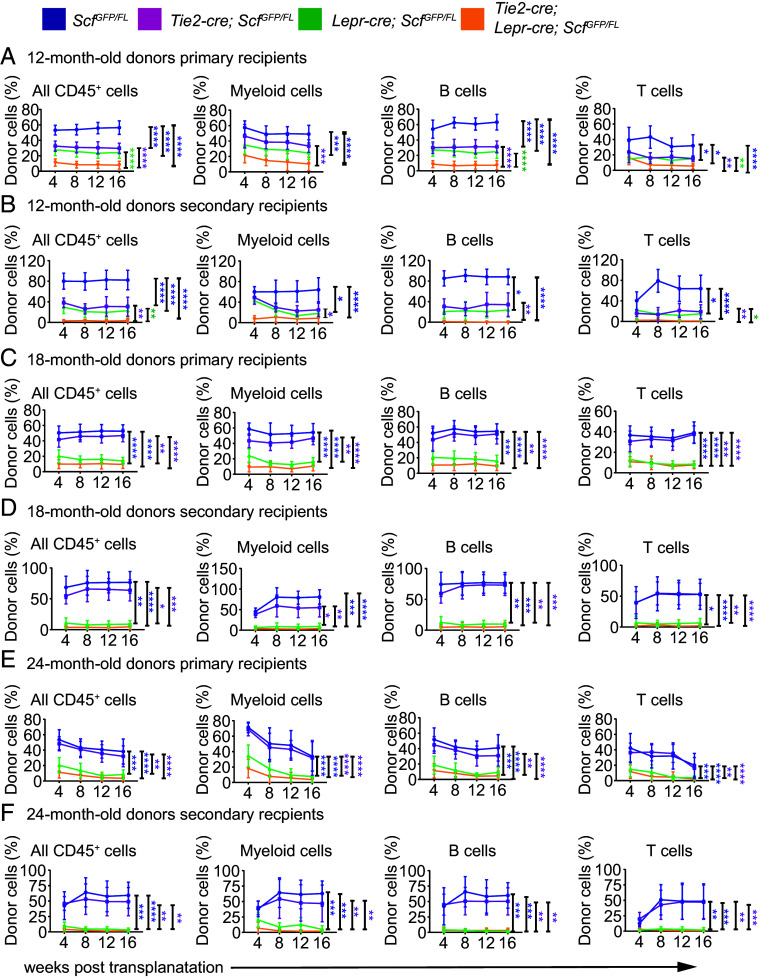
*Scf* deletion from *Lepr^+^* stromal cells decreased the reconstituting capacity of whole bone marrow cells upon competitive transplantation into irradiated mice. (*A*) Donor cell contributions to CD45^+^ cells as well as myeloid, B, and T cells in the blood of mice competitively transplanted with donor bone marrow cells from 12-mo-old mice. (*B*) Secondary recipients of bone marrow cells from the mice in (*A*). (*C*) Donor cell contributions to the blood of mice competitively transplanted with donor bone marrow cells from 18-mo-old mice. (*D*) Secondary recipients of bone marrow cells from the mice in (*C*). (*E*) Donor cell contributions to the blood of mice competitively transplanted with donor bone marrow cells from 24-mo-old mice. (*F*) Secondary recipients of bone marrow cells from the mice in (*E*). In each of the primary transplants shown above, three donors per genotype were transplanted into a total of 14 to 15 recipients per genotype in three independent experiments. In each of the secondary transplants shown above, three donors per genotype were transplanted into a total of 10 secondary recipients per genotype in three independent experiments. All data represent mean ± SD (*P < 0.05; **P < 0.01; ***P < 0.001; ****P < 0.0001).

To test whether *Lepr*^+^ cells and endothelial cells remain functionally important sources of *Scf* for HSC and restricted hematopoietic progenitor maintenance in aging bone marrow, we aged *Tie2-cre; Scf^GFP/FL^* mice, *Lepr-cre; Scf^GFP/FL^* mice, and *Tie2-cre; Lepr-cre; Scf^GFP/FL^* mice. We assessed the numbers of HSCs, MPPs, and several restricted hematopoietic progenitors by flow cytometry in the bone marrow of 12-, 18-, and 24-mo-old mice. The gating strategy for identifying each of the hematopoietic stem and progenitor cell populations is shown in *SI Appendix*, Fig. S6. *Tie2-cre; Scf^GFP/FL^* and *Scf^GFP/FL^* control mice did not significantly differ in terms of white blood cell (WBC), red blood cell (RBC), or platelet counts at 12, 18, or 24 mo of age (Fig. 2 *A*–*C*), bone marrow (Fig. 2*D*) or spleen (Fig. 2*E*) cellularity, or the number of HSCs in the spleen (Fig. 2*F*). HSC numbers were significantly reduced in the bone marrow of *Tie2-cre; Scf^GFP/FL^* as compared to *Scf^GFP/FL^* mice at 12 mo of age (Fig. 2*G*) but not at 18 or 24 mo of age (Fig. 2*G*). Consistent with this, bone marrow competitively transplanted from *Tie2-cre; Scf^GFP/FL^* mice gave significantly lower levels of donor cell reconstitution as compared to *Scf^GFP/FL^* bone marrow at 12 mo of age, but not at 18 or 24 mo of age in primary (Fig. 3 *A*, *C*, and *E*) and secondary recipient mice (Fig. 3 *B, D*, and *F*). All of the primary and secondary recipients were long-term multilineage reconstituted by donor cells (*SI Appendix*, Table S3). Endothelial cells are thus a functionally important source of SCF for HSC maintenance in young adult bone marrow but not in old adult bone marrow (18 and 24 mo of age).

In contrast to endothelial cells, LepR^+^ cells were an important source of SCF for HSC maintenance at all ages. *Lepr-cre; Scf^GFP/FL^* mice had significantly reduced WBC, RBC, and PLT counts as compared to *Scf^GFP/FL^* controls at 12, 18, and 24 mo of age (with the exception of WBC counts at 12 mo of age) (Fig. 2 *A*–*C*). Bone marrow cellularity (Fig. 2*D*) and the number of HSCs in bone marrow (Fig. 2*G*) were also significantly reduced in *Lepr-cre; Scf^GFP/FL^* as compared to *Scf^GFP/FL^* mice at 12, 18, and 24 mo of age. Consistent with this, spleen cellularity and the number of HSCs in the spleen were significantly increased in *Lepr-cre; Scf^GFP/FL^* as compared to *Scf^GFP/FL^* mice at 12, 18, and 24 mo of age (Fig. 2 *E* and *F*). Genetic changes that deplete HSCs from the bone marrow lead to extramedullary hematopoiesis, increasing cellularity and HSC frequency in the spleen (9). Finally, competitive transplantation assays showed that bone marrow cells from *Lepr-cre; Scf^GFP/FL^* mice gave significantly lower levels of donor cell reconstitution than *Scf^GFP/FL^* bone marrow cells at 12, 18, and 24 mo of age in primary (Fig. 3 *A*, *C*, and *E*) and secondary recipient mice (Fig. 3 *B*, *D*, and *F*). All of the primary and secondary recipients of 12- and 18-mo-old *Lepr-cre; Scf^GFP/FL^* bone marrow were long-term multilineage reconstituted by donor cells but most of the secondary recipients of 24-mo-old *Lepr-cre; Scf^GFP/FL^* bone marrow were only transiently reconstituted by donor cells (*SI Appendix*, Table S3). SCF from LepR^+^ stromal cells is thus required to maintain normal numbers of HSCs in aging bone marrow.

Consistent with the results above, white blood cell counts (Fig. 2*A*) and the number of HSCs in the bone marrow (Fig. 2*G*) were significantly reduced at 12 mo of age in *Tie2-cre; Lepr-cre; Scf^GFP/FL^* (deleting in both endothelial cells and *Lepr*^+^ cells) as compared to *Lepr-cre; Scf^GFP/FL^* mice (deleting only in *Lepr*^+^ cells). At 18 and 24 mo of age, almost no hematopoietic parameters significantly differed between *Tie2-cre; Lepr-cre; Scf^GFP/FL^* and *Lepr-cre; Scf^GFP/FL^* mice (Fig. 2 *A*–*G*). The only exception was that the number of HSCs was significantly reduced in the bone marrow of *Tie2-cre; Lepr-cre; Scf^GFP/FL^* as compared to *Lepr-cre; Scf^GFP/FL^* mice at 24, but not at 18, mo of age (Fig. 2*G*). This raised the possibility that endothelial cells produce enough SCF to partially compensate for the loss of SCF from *Lepr*^+^ cells in aging bone marrow. However, competitive transplantation assays showed that *Tie2-cre; Lepr-cre; Scf^GFP/FL^* bone marrow cells gave significantly lower levels of donor cell reconstitution as compared to *Lepr-cre; Scf^GFP/FL^* bone marrow cells at 12 mo of age (Fig. 3*A*) but not at 18 (Fig. 3*C*) or 24 (Fig. 3*E*) mo of age. These observations are consistent with the finding that both endothelial cells and LepR^+^ cells contributed SCF to HSC maintenance at 12 mo of age but we did not detect a clear effect of SCF from endothelial cells at 18 or 24 mo of age.

### SCF from LepR^+^ Cells Maintains Restricted Progenitors During Aging.

**Fig. 4.**
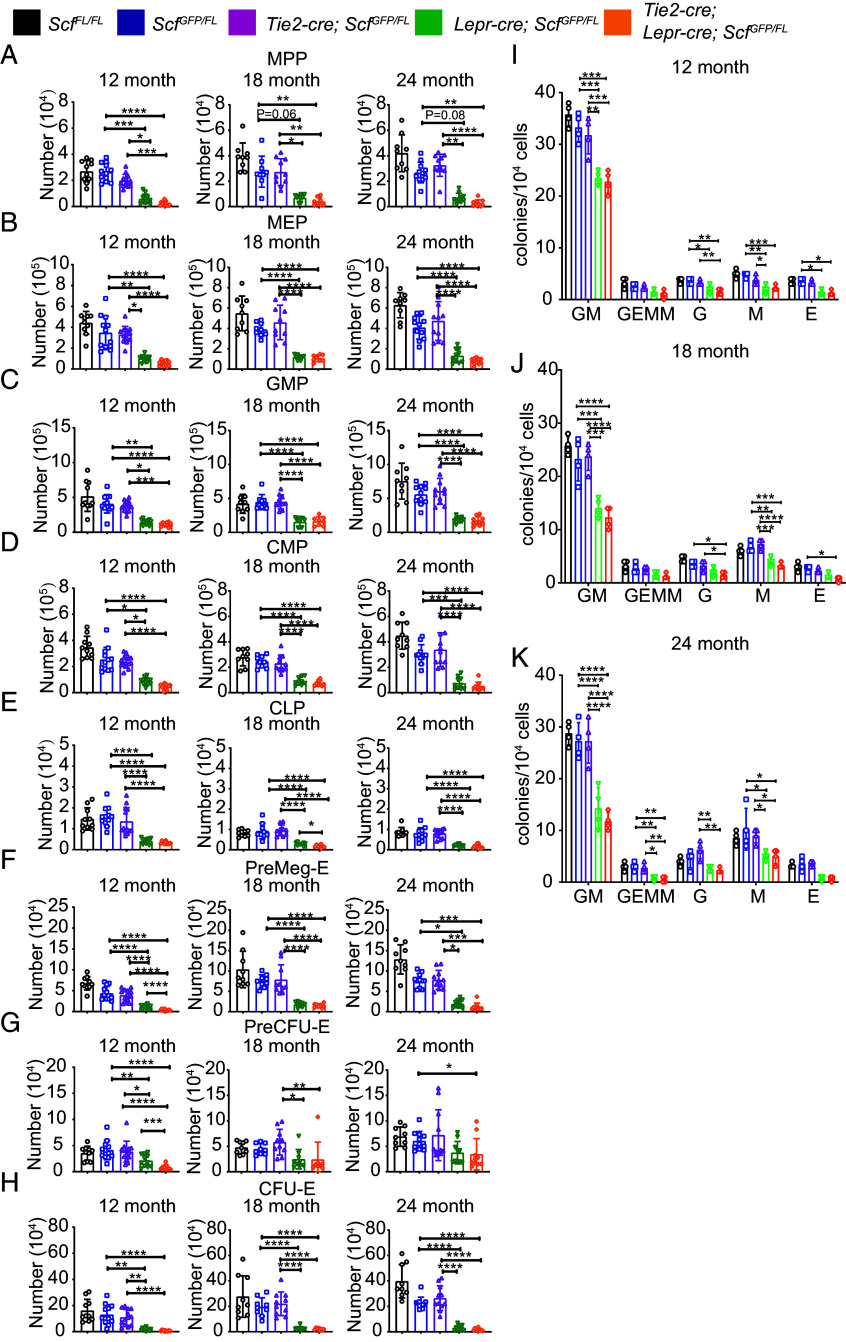
*Scf* deletion from *Lepr^+^* stromal cells depleted several c-kit^+^ restricted hematopoietic progenitors in the bone marrow of 12-, 18-, and 24-mo-old mice. (*A–H*) Numbers of MPPs (*A*), MEPs (*B*), GMPs (*C*), CMPs (*D*), CLPs (*E*), pre-MegEs (*F*), pre-CFU-Es (*G*), and CFU-Es (*H*) in the bone marrow of 12-, 18-, 24- mo-old mice. (*I–K*) The numbers of colonies formed by 10,000 bone marrow cells from 12 (*I*), 18 (*J*), and 24 (*K*) mo-old mice (G, granulocyte; M, macrophage; E, erythrocyte-containing colonies). Each dot represents a different mouse. In *A*–*H*, each panel shows 10 to 13 mice per genotype in six independent experiments for 12-mo-old mice, 8 to 10 mice per genotype in five experiments for 18-mo-old mice, and 9 to 11 mice per genotype in six experiments for 24-mo-old mice. All data represent mean ± SD (*P < 0.05; **P < 0.01; ***P < 0.001; ****P < 0.0001).

We assessed the numbers of multipotent progenitors (MPPs) and restricted hematopoietic progenitors in the bone marrow of *Tie2-cre; Scf^GFP/FL^* mice, *Lepr-cre; Scf^GFP/FL^* mice, and *Tie2-cre; Lepr-cre; Scf^GFP/FL^* mice at 12, 18, and 24 mo of age. *Tie2-cre; Scf^GFP/FL^* and *Scf^GFP/FL^* control mice did not significantly differ in terms of the number of any multipotent or restricted progenitor cell population we examined including MPPs (Fig. 4*A*), megakaryocyte-erythroid progenitors (MEPs) (Fig. 4*B*), granulocyte-monocyte progenitors (GMPs) (Fig. 4*C*), common myeloid progenitors (CMPs) (Fig. 4*D*), common lymphoid progenitors (CLPs) (Fig. 4*E*), pre-megakaryocyte-erythroid progenitors (preMegEs) (Fig. 4*F*), pre-colony-forming units-erythrocyte (preCFU-Es) (Fig. 4*G*), or CFU-E (Fig. 4*H*). The markers and flow cytometry gates used to identify each cell population are shown in *SI Appendix*, Table S2 and Fig. S6, respectively. SCF from endothelial cells was thus not required for the maintenance of any restricted hematopoietic progenitor cell population at 12, 18, or 24 mo of age.

In contrast, LepR^+^ cells were a functionally important source of SCF for the maintenance of all of the multipotent and restricted progenitor cell populations that we analyzed at all ages with the exception of MPPs (Fig. 4*A*; *P* = 0.06 and 0.08 at 18 and 24 mo of age, respectively) and preCFU-Es (Fig. 4*G*) at 18 mo and 24 mo of age. *Lepr-cre; Scf^GFP/FL^* mice had significantly fewer MEPs (Fig. 4*B*), GMPs (Fig. 4*C*), CMPs (Fig. 4*D*), CLPs (Fig. 4*E*), preMegEs (Fig. 4*F*), and CFU-Es (Fig. 4*H*) in the bone marrow as compared to *Scf^GFP/FL^* control mice at 12, 18, and 24 mo of age. Consistent with this, *Tie2-cre; Lepr-cre; Scf^GFP/FL^* and *Lepr-cre; Scf^GFP/FL^* mice did not significantly differ in the numbers of most multipotent and restricted progenitor cell populations in the bone marrow (Fig. 4 *A*–*H*). The exceptions were that *Tie2-cre; Lepr-cre; Scf^GFP/FL^* mice had significantly fewer preMegEs (Fig. 4*F*) and preCFU-Es (Fig. 4*G*) at 12 mo of age and CLPs at 18 mo of age (Fig. 4*E*).

We also assessed the frequencies of restricted progenitors using colony-forming assays. Again, *Tie2-cre; Scf^GFP/FL^* and *Scf^GFP/FL^* control mice did not significantly differ in the frequencies of any colony-forming progenitor, including CFU-GM, CFU-GEMM, CFU-G, CFU-M, or CFU-E (Fig. 4 *I*–*K*). *Lepr-cre; Scf^GFP/FL^* mice generally had significantly lower frequencies of CFU-GM and CFU-M as compared to *Scf^GFP/FL^* controls at 12, 18, and 24 mo of age (Fig. 4 *I*–*K*). *Lepr-cre; Scf^GFP/FL^* mice also had significantly lower frequencies of CFU-E at 12 mo of age (Fig. 4*I*) and CFU-GEMM at 24 mo of age (Fig. 4*K*). Finally, *Tie2-cre; Lepr-cre; Scf^GFP/FL^* mice had significantly lower frequencies of CFU-GM, CFU-G, CFU-M, and CFU-E as compared to *Scf^GFP/FL^* mice at most ages (Fig. 4 *I*–*K*). LepR^+^ cells were thus the key source of SCF for the maintenance of most colony-forming progenitors at 12, 18, and 24 mo of age.

### The Periarteriolar Environment Becomes Inflamed During Aging.

**Fig. 5.**
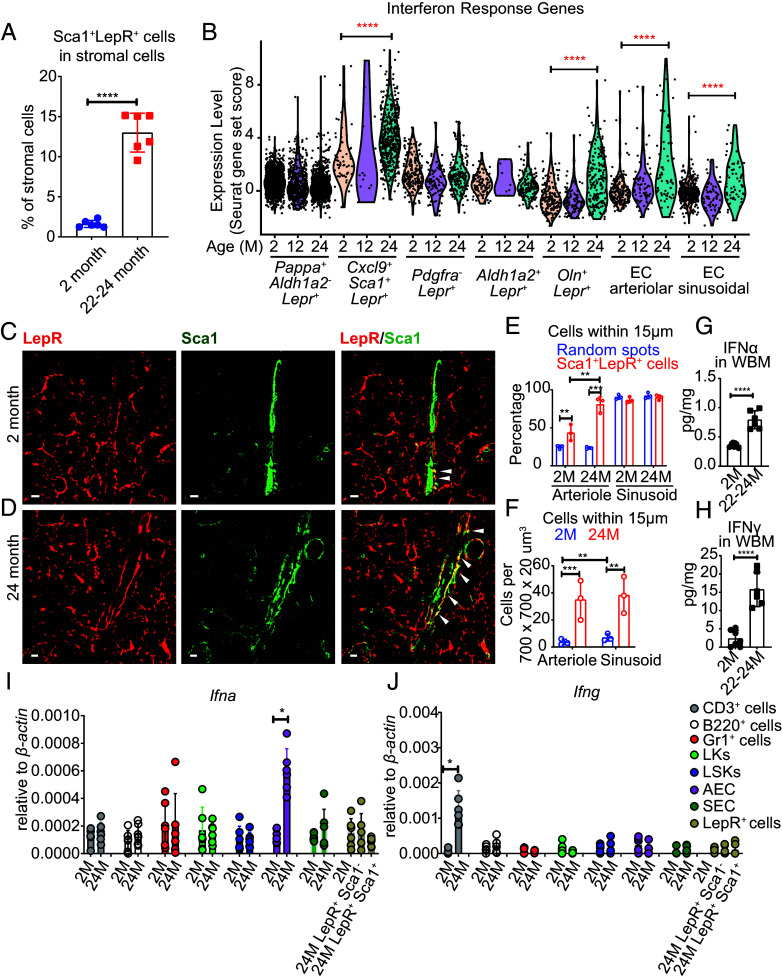
Increasing interferon levels during aging induce interferon-regulated gene expression by periarteriolar stromal cells in the bone marrow. (*A*) The frequency of Sca1^+^LepR^+^ stromal cells increased approximately 10-fold during aging in the bone marrow (six mice per time point from two independent experiments). (*B*) Expression of interferon-regulated genes in *Lepr^+^* cell and endothelial cell clusters. These data reflect 40 interferon-regulated genes from the single-cell RNA sequencing data in Fig. 1. The individual genes are shown in *SI Appendix*, Fig. S8*A*. (*C* and *D*) Anti-LepR and anti-Sca1 antibody staining in femur bone marrow sections from 2 (*C*) and 24 (*D*) mo-old mice shows that periarteriolar Sca1^+^LepR^+^ cells (arrowheads) increased in frequency with age. The Sca1^+^LepR^+^ cells were adjacent to Sca1^+^ arteriolar endothelial cells. (Scale bar, 10 µm.) (*E*) The percentages of Sca1^+^LepR^+^ cells and random spots that were within 15 μm of arterioles and sinusoids in thick femur sections from mice at 2 mo or 24 mo of age (n = 3). (*F*) The numbers of Sca1^+^LepR^+^ cells that were within 15 μm of arterioles and sinusoids in thick femur sections from mice at 2 mo or 24 mo of age (n = 3). (*G* and *H*) IFNα (*G*) and IFNγ (*H*) levels by ELISA in bone marrow lysate from 2- and 22 to 24-mo-old mice (n = 6 to 7 mice per time point from two independent experiments). (*I* and *J*) *Ifna* and *Ifng* transcript levels by qRT-PCR in CD3^+^ T cells, B220^+^ B cells, Gr1^+^ myeloid cells, LK myeloid progenitors, LSK stem/progenitor cells, arteriolar endothelial cells, sinusoidal endothelial cells, and LepR^+^ stromal cells from the bone marrow of 2- and 24-mo-old mice (n = 6 mice per time point from two independent experiments). Each dot represents a different mouse in panels *A* and *E–J*. All data represent mean ± SD (*P < 0.05; **P < 0.01; ***P < 0.001; ****P < 0.0001).

Given that LepR^+^ cells remained the key source of SCF for the maintenance of HSCs and restricted hematopoietic progenitors at all ages, we assessed whether these cells underwent changes during aging. Based on single-cell RNA sequencing, the stromal cell population that changed most in frequency with age was the *Cxcl9^+^Sca1^+^Lepr^+^* cell cluster, which represented around 2% of bone marrow stromal cells at 2 and 12 mo of age, but 22% at 24 mo of age (*SI Appendix*, Table S1). *Cxcl9^+^Sca1^+^Lepr^+^* cells were unique among *Lepr^+^* cell clusters in expressing Sca1 (encoded by the *Ly6a* gene; Fig. 1*C*). By flow cytometry, Sca-1^+^LepR^+^ cells represented 1.6 ± 0.4% of stromal cells in 2-mo-old bone marrow and 13 ± 2.4% of stromal cells in 22 to 24-mo-old bone marrow (Fig. 5*A*). The frequency and absolute number of LepR^+^ cells in the bone marrow did not significantly change with age (*SI Appendix*, Fig. S3 *A* and *B*) and we did not observe a significant increase in the expression of cell cycle genes in *Cxcl9^+^Sca1^+^Lepr^+^* cells during aging (*SI Appendix*, Fig. S5). It remains possible that cell division contributed to the increase in the number of *Cxcl9^+^Sca1^+^Lepr^+^* cells during aging; however, *Cxcl9^+^Sca1^+^Lepr^+^* cells may have increased in number during aging as a result of conversion from other subsets of LepR^+^ cells. *Pappa^+^Aldh1a2^−^Lepr^+^* cells declined in frequency during aging (*SI Appendix*, Table S1) and both Cxcl9 and Sca1 are interferon-regulated genes (54–56). This raised the possibility that interferon converted other subsets of LepR^+^ cells into *Cxcl9^+^Sca1^+^Lepr^+^* cells by inducing Cxcl9 and Sca1 expression.

All 8 of the most highly enriched GO terms in *Cxcl9^+^Sca1^+^Lepr^+^* cells as compared to other *Lepr^+^* cell clusters related to interferon-regulated genes (*SI Appendix*, Fig. S4*B*), including *Cxcl9*, *Stat1*, and *Sca1* (*Ly6a*), all of which were more highly expressed in *Cxcl9^+^Sca1^+^Lepr^+^* cells as compared to other stromal cells (Fig. 1*C*). To further examine this, we assembled a list of 40 interferon-regulated genes from these GO terms that were detected in our single-cell RNA sequencing analysis and compared the average expression levels in each cell cluster at 2, 12, and 24 mo of age. The average expression levels increased significantly with age in *Cxcl9^+^Sca1^+^Lepr^+^* cells (Fig. 5*B*). Both type 1 and type 2 interferon receptors (*Ifnar2* and *Ifngr1*) were broadly expressed by all clusters of LepR^+^ cells and endothelial cells but not by osteoblasts or chondrocytes (*SI Appendix*, Fig. S7 *A*–*D*). We also observed significant increases in the expression of interferon-regulated genes with age in *Osteolectin^+^Lepr^+^* cells, arteriolar endothelial cells, and sinusoidal endothelial cells, but to a lesser extent than in *Cxcl9^+^Sca1^+^Lepr^+^* cells (Fig. 5*B*). The most profound increases in interferon-regulated genes during aging were in *Cxcl9^+^Sca1^+^Lepr^+^* cells and arteriolar endothelial cells (*SI Appendix*, Fig. S8*A*).

To test whether *Cxcl9^+^Sca1^+^Lepr^+^* cells were periarteriolar, we stained bone marrow sections with antibodies against Sca1 and LepR. Sca1 is expressed by both arteriolar endothelial cells (57) and *Cxcl9^+^Sca1^+^Lepr^+^* cells (Fig. 1*C*). We observed Sca1^+^LepR^−^ endothelial cells (green) in arterioles and Sca1^+^LepR^+^ stromal cells (green and red = yellow) surrounding these endothelial cells (Fig. 5 *C* and *D*). Sca1^+^LepR^+^ cells were significantly more likely than random spots to localize within 15 µm of arterioles at 2 and 24 mo of age, and the numbers of Sca1^+^LepR^+^ cells that localized within 15 µm of arterioles was significantly higher at 24 as compared to 2 mo of age (Fig. 5 *E* and *F*): 81 ± 10% of Sca1^+^LepR^+^ cells were periarteriolar at 24 mo of age (Fig. 5*E*). The percentage of Sca1^+^LepR^+^ cells that localized within 15 µm of sinusoids did not significantly differ from random spots at 2 or 24 mo of age and did not change with age (Fig. 5*E*). Consistent with the increase in the numbers of Sca1^+^LepR^+^ cells in the bone marrow of old as compared to young mice (Fig. 5 *A*, *C*, and *D*), we also observed significant increases in the number of Sca1^+^LepR^+^ cells within 15 µm of arterioles and sinusoids in the bone marrow of 24-mo-old as compared to 2-mo-old mice (Fig. 5*F*). Since sinusoids are ubiquitous throughout the bone marrow, many of the Sca1^+^LepR^+^ cells near arterioles were also near sinusoids. Nonetheless, since Sca1^+^LepR^+^ cells were significantly more likely than random spots to localize near arterioles, but not sinusoids, we consider most Sca1^+^LepR^+^ cells to be periarteriolar.

In light of the increased expression of interferon-regulated genes during aging, particularly in periarteriolar stromal cells, we assessed interferon levels in blood serum and bone marrow by enzyme-linked immunosorbent assay (ELISA). We observed significantly increased levels of IFNα and IFNγ in the bone marrow of 22 to 24-mo-old mice as compared to 2-mo-old mice (Fig. 5 *G* and *H*). We also observed increased levels of IFNγ, but not IFNα, in the serum of 22 to 24-mo-old mice as compared to 2-mo-old mice (*SI Appendix*, Fig. S8 *B* and *C*). We did not observe any changes in IFNβ levels in the bone marrow or serum (*SI Appendix*, Fig. S8 *D* and *E*).

Since interferon expression is rarely detected by RNA sequencing, we performed quantitative RT-PCR on bone marrow hematopoietic cells and stromal cells to identify cells that produced interferons during aging. In most cell populations, we did not detect any increase in the expression of *Ifna* or *Ifng*, including B220^+^ B cells, Gr1^+^ myeloid cells, Lineage^−^c-kit^+^ myeloid progenitors, LSK stem/progenitor cells, sinusoidal endothelial cells, or LepR^+^ cells (Fig. 5 *I* and *J*). However, there were two bone marrow cell populations that did show an increase in *Ifn* expression during aging: *Ifna* expression by arteriolar endothelial cells (Fig. 5*I*) and *Ifng* expression by CD3^+^ T cells (Fig. 5*J*). *Ifnb* expression did not change during aging (*SI Appendix*, Fig. S8 *F* and *G*).

**Fig. 6.**
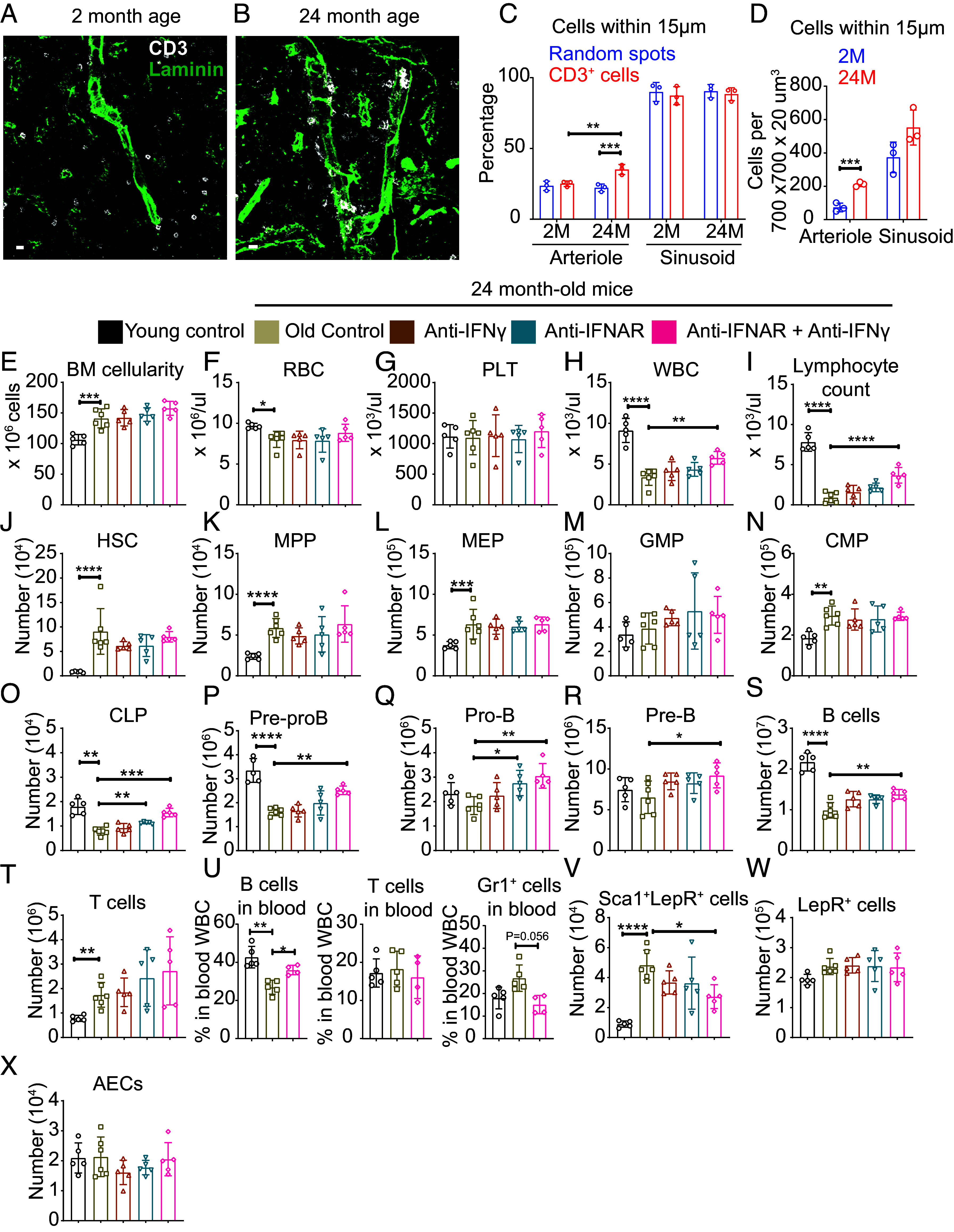
Blocking interferon signaling partially rescued the decline in lymphopoiesis and the increase in the frequency of Sca1^+^LepR^+^ stromal cells in aging bone marrow. (*A* and *B*) Representative CD3 and laminin staining in bone marrow sections from 2 (*A*) and 24 (*B*) mo-old mice. (Scale bar, 10 µm.) These images show increased clustering of CD3^+^ T cells (white) around arterioles in old bone marrow (images are representative of three mice per time point). (*C*) The percentages of CD3^+^ cells and random spots that were within 15 μm of arterioles and sinusoids in thick femur sections from mice at 2 mo or 24 mo of age (n = 3). (*D*) The numbers of CD3^+^ cells that were within 15 μm of arterioles and sinusoids in thick femur sections from mice at 2 mo or 24 mo of age (n = 3). (*E*) Cellularity of two femurs and two tibias from 2-mo-old control mice, 24-mo-old control mice, and 24-mo-old mice treated with anti-IFNγ antibody alone, anti-IFNAR antibody alone, or the combination of anti-IFNγ plus anti-IFNAR antibodies. (*F–I*) RBC (*F*), PLT (*G*), WBC (*H*), and lymphocyte counts (*I*) in the blood of the same mice. (*J–T*) The numbers of HSCs (*J*), MPPs (*K*), MEPs (*L*), GMPs (*M*), CMPs (*N*), CLPs (*O*), Pre-proB cells (*P*), Pro-B cells (*Q*), Pre-B cells (*R*), B220^+^ B cells (*S*), and T cells (*T*) in the bone marrow. (*U*) The percentages of WBCs that were B, T, or Gr1^+^ cells. (*V–X*) The numbers of Sca1^+^LepR^+^ stromal cells (*V*), total LepR^+^ stromal cells (*W*), and arteriolar endothelial cells (AECs) (*X*) in the bone marrow. Each dot represents a different mouse in panels *E–X*. The data in panels *E–X* represent a total of four to six mice per treatment from two independent experiments. All data represent mean ± SD (*P < 0.05; **P < 0.01; ***P < 0.001; ****P < 0.0001).

A subset of CD3^+^ T cells was closely associated with arterioles in the bone marrow (Fig. 6 *A* and *B*). CD3^+^ cells were significantly more likely than random spots to localize within 15 µm of arterioles at 24, but not at 2, mo of age, and the percentage of CD3^+^ cells that localized within 15 µm of arterioles was significantly higher at 24 as compared to 2 mo of age (Fig. 6*C*): 35 ± 3.5% of CD3^+^ cells were periarteriolar at 24 mo of age. The percentage of CD3^+^ cells that localized within 15 µm of sinusoids did not significantly differ from random spots at 2 or 24 mo of age and did not change with age. Consistent with the increase in the numbers of CD3^+^ cells in the bone marrow of old as compared to young mice (Fig. 6*T*), we also observed a significant increase in the absolute number of CD3^+^ cells within 15 µm of arterioles in the bone marrow of 24-mo-old as compared to 2-mo-old mice (Fig. 6*D*). It is possible that *Cxcl9^+^Sca-1^+^Lepr^+^* cells recruit T cells to the arterioles during aging by expressing increasing levels of *Cxcl9* though at present we do not have the genetic tools required to test this functionally. Cxcl9 is an interferon-induced chemokine (54, 55) that promotes T cell recruitment to sites of inflammation (58). Consistent with a prior study (14), *Il7* was mainly expressed by *Lepr^+^* cells and its expression did not significantly change with age (*SI Appendix*, Fig. S8*I*). These results suggest that an inflammatory microenvironment develops around arterioles during aging marked by interferon expression in arteriolar endothelial cells and an interferon response in *Cxcl9^+^Sca1^+^Lepr^+^* cells. Nonetheless, IFNγ levels were also increased in the serum of old as compared to young mice and it is not clear whether IFNγ in the blood would preferentially induce interferon responses in periarteriolar cells as compared to perisinusoidal cells.

### Interferon Promotes Lymphoid Progenitor Depletion During Aging.

We tested whether inhibition of interferon signaling would rescue the depletion of lymphoid progenitors during aging. We treated 24-mo-old mice with blocking antibodies against the type 1 interferon receptor (IFNAR, which binds IFNα and IFNβ) and/or IFNγ for 3 wk. Treatment with individual antibodies did not significantly alter most hematopoietic parameters as compared to untreated control mice (Fig. 6 *E*–*T*, except that anti-IFNAR treatment alone increased the number of CLPs (Fig. 6*O*) and Pro-B cells in bone marrow of old mice (Fig. 6*Q*). CLPs express the type 1 and type 2 interferon receptors (59) and exhibit an increase in interferon-regulated gene expression during aging (60). Treatment with the combination of anti-IFNAR and anti-IFNγ did not significantly alter most hematopoietic parameters but did significantly increase white blood cell counts (Fig. 6*H*), lymphocyte counts in the blood (Fig. 6*I*), and the numbers of CLPs, Pre-proB cells, Pro-B cells, Pre-B cells, and total B cells in bone marrow (Fig. 6 *O*–*S*) relative to untreated 24-mo-old control mice. These data suggest that treatment with blocking antibodies against IFNAR and IFNγ partially rescued the decline in B lymphopoiesis during aging. Treatment with the combination of anti-IFNAR and anti-IFNγ did not influence the number of T cells in bone marrow (Fig. 6*T*). Consistent with this, the combination of anti-IFNAR and anti-IFNγ antibodies increased the percentage of WBCs that were B cells, but not T cells or Gr1^+^ cells (Fig. 6*U*). Treatment with the combination of anti-IFNAR and anti-IFNγ also significantly decreased the numbers of Sca1^+^LepR^+^ stromal cells in the bone marrow of old mice (Fig. 6*V*), but did not significantly alter the total number of LepR^+^ cells (Fig. 6*W*) or arteriolar endothelial cells (Fig. 6*X*). Treatment with the combination of anti-IFNAR and anti-IFNγ significantly decreased the expression of *Cxcl9*, an interferon-regulated gene (54, 55), by Sca1^+^LepR^+^ stromal cells from old mice (*SI Appendix*, Fig. S8*H*). The increased levels of interferon in old mice, thus, increased the numbers of Sca1^+^LepR^+^ stromal cells in the bone marrow and contributed to the decline in lymphopoiesis during aging.

## Discussion

In this study, we show that LepR^+^ cells remain the major source of *Scf* and *Cxcl12* at all ages in adult bone marrow, including during aging (Fig. 1 *D* and *E*). The SCF produced by LepR^+^ cells remained functionally important for the maintenance of HSCs and restricted hematopoietic progenitors in old bone marrow (Figs. 2–4). In contrast, SCF produced by endothelial cells contributed to HSC maintenance in the bone marrow of 12-mo-old mice but had no clear effect at 18 or 24 mo of age (Figs. 2 and 3).

By single-cell RNA sequencing, we identified five transcriptionally distinct subsets of LepR^+^ cells, including *Sca1^+^Cxcl9^+^LepR^+^* periarteriolar stromal cells (Fig. 1*B*) that expanded in number by 10-fold during aging (Fig. 5*A*) and that were marked by increasing expression of interferon-regulated genes during aging (Fig. 5*B*). The periarteriolar microenvironment in the bone marrow exhibited increased expression of *Ifna* by arteriolar endothelial cells (Fig. 5*I*) and an increasing percentage of CD3^+^ T cells clustered around arterioles (Fig. 6 *B*–*D*) during aging. Interferon contributed to the expansion in the number of *Sca1^+^Cxcl9^+^LepR^+^* cells during aging as well as the depletion of lymphoid progenitors as both phenotypes were partially rescued by treating old mice with blocking antibodies against IFNAR and IFNγ (Fig. 6). However, it remains unclear whether interferons acted directly on lymphoid progenitors or whether the effects were mediated by changes in stromal cells.

The levels of multiple inflammatory cytokines increase during aging in the bone marrow, including CCL5 (28, 29), IL1b (25), IL6 (27, 30), and TNF (27, 30–33). However, we did not detect significant expression of these factors by single-cell RNA sequencing in any bone marrow stromal cell population. Some of these factors are expressed by hematopoietic cells (61, 62). It is also possible that some of these factors are not readily detected by single-cell RNA sequencing. In any case, functional studies have shown that these factors contribute to the increase in myelopoiesis in aging bone marrow as well as the decline in osteogenesis (25, 29, 63, 64). In addition to these increases in inflammatory cytokines, there is also a decline in factors that promote HSC function during aging, including IGF1 (33).

Our results suggest that aging-associated inflammation may not be uniformly distributed in the bone marrow, as arteriolar endothelial cells and periarteriolar *Sca1^+^Cxcl9^+^LepR^+^* stromal cells exhibit the strongest interferon-regulated gene signatures among stromal cells in the bone marrow. An interesting question for future studies is whether other inflammatory cytokines are also spatially limited in their expression or action within aging bone marrow.

## Materials and Methods

### Mice.

*Scf^FL^* mice (*Kitltm2.1Sjm/J*, RRID:IMSR_JAX:017861), *Scf^GFP^* mice (*Kitltm1.1Sjm/J*, RRID:IMSR_JAX:017860), and *Cxcl12^dsred^* mice (*Cxcl12tm2.1Sjm/J*, RRID:IMSR_JAX:022458) were generated in our laboratory (9, 10). *Tie2-cre* mice (*B6.Cg-Tg(Tek-cre)1Ywa/J*, RRID:IMSR_JAX:008863) (65) and *Lepr-cre* mice (*B6.129(Cg)-Leprtm2(cre)Rck/J*, RRID:IMSR_JAX:008320) (66) were obtained from Jackson Laboratory. All mice were backcrossed at least six times onto a C57BL/Ka background. We aged male and female mice to 12, 18, or 24 mo old and used these mice for experiments. All mice were housed in AAALAC-accredited, specific-pathogen-free animal care facilities at the University of Texas Southwestern Medical Center (UTSW). Mice were housed under a 12 h:12 h light:dark cycle with a temperature of 18 to 24 °C and humidity of 35 to 60% and were fed Teklad Global 16% Protein Rodent Diet ad libitum. All procedures were approved by the UTSW Institutional Animal Care and Use Committee (5, 52).

### Flow Cytometric Analysis of Hematopoietic Cells.

Tibias and femurs were crushed, triturated, and resuspended in staining medium [Ca^2+^ and Mg^2+^ free Hank’s buffered salt solution (HBSS) supplemented with 3% heat-inactivated bovine serum] and filtered through a 40 μm cell strainer (Fisher Scientific) to generate a single-cell suspension of bone marrow cells as previously described (5, 52). Spleens were also filtered through a 40 μm cell strainer to generate a single-cell suspension. Cells were counted and then stained with antibodies at 4 °C for 30 min. For staining of HSCs, MPPs, and HPCs, cells were stained with fluorophore-conjugated antibodies against c-kit, Sca1, CD150, CD48, and lineage markers (CD2, CD3, CD5, CD8a, B220, Ter119, and Gr-1). For staining of restricted hematopoietic progenitors, cells were stained with fluorophore-conjugated antibodies against c-kit, Sca1, CD150, CD48, CD105, CD41, CD34, CD16/32, and lineage markers (CD2, CD3, CD5, CD8a, B220, Ter119, and Gr-1). For analysis of lymphoid and myeloid cells, cells were stained with fluorophore-conjugated antibodies against B220, CD3, Gr-1, Mac1, and Ter119. After staining with antibodies, the cells were washed and resuspended in staining medium with 2 µg/mL of DAPI (to discriminate live from dead cells) to exclude dead cells. The cells were then sorted or analyzed using a FACSAsria II (BD Bioscience) or a FACSAria Fusion (BD Bioscience) flow cytometer. Markers for each hematopoietic cell population are listed in *SI Appendix*, Table S2.

### Flow Cytometric Analysis of Bone Marrow Stromal Cells.

To isolate bone marrow stromal cells, whole femurs and tibias were crushed, then the bone marrow was collected and digested in enzymatic dissociation buffer [HBSS with Ca^2+^ and Mg^2+^ (Corning), supplemented with 200 U/mL Dnase I (Roche), 4 mg/mL Dispase (Sigma-Aldrich), and 3 mg/mL Collagenase type 1 (Worthington Biochemical)] at 37 °C for 30 min as described (2). After a brief vortex at medium speed, the samples sedimented for 1 min, then the cell suspension was transferred to a new tube containing 10 mL of staining medium (Ca^2+^ and Mg^2+^ free HBSS containing 3% heat-inactivated bovine serum) with 2 mM EDTA on ice. We then centrifuged at 500 × g for 5 min at 4 °C and resuspended the cell pellet in staining medium. Cells were then incubated with biotin-conjugated anti-LepR antibody (R&D Systems) for 1.5 h at 4 °C. Cells were then washed and incubated with fluorophore-conjugated antibodies against CD45 (eBioscience), Ter119 (eBioscience), Sca1 (eBioscience), and APC-conjugated streptavidin (BioLegend) for 30 min at 4 °C. Cells were washed again and resuspended in staining medium containing 2 µg/mL DAPI for flow cytometric analysis or sorting using a FACSAria II (BD Bioscience) or a FACSAria Fusion (BD Bioscience).

### Additional Methods Are in *SI Appendix*.

Additional materials and methods can be found in **SI Appendix*, *Materials and Methods**.

## Supplementary Material

Appendix 01 (PDF)

## Data Availability

Raw RNA sequencing data generated in this study are available in the BioProject database under accession number PRJNA1085191 (67). All other data are included in the manuscript and/or *SI Appendix*.
