# Supplementary material for: Periarteriolar niches become inflamed in aging bone marrow, remodeling the stromal microenvironment and depleting lymphoid progenitors
Source: Proc Natl Acad Sci U S A. 2025 Mar 10;122(11):e2412317122. doi: 10.1073/pnas.2412317122 (PMC11929388; doi:10.1073/pnas.2412317122)
Supplement: Supplementary file 1 — Appendix 01 (PDF) [file pnas.2412317122.sapp.pdf]

Supporting Information for

Periarteriolar niches become inflamed in aging bone marrow, remodeling the stromal microenvironment and depleting lymphoid progenitors

Liming Du[1, 2], Maria Angelica Freitas-Cortez[1], Jingzhu Zhang[1,3], Yuanyuan Xue[1], Reshma T. Veettil[1], Zhiyu Zhao[1], Sean J. Morrison[1,4,*]

[1] Children's Research Institute and the Department of Pediatrics, University of Texas Southwestern Medical Center, Dallas, TX 75390, USA

[2] Current Address: Shandong Provincial Key Laboratory of Animal Cell and Developmental Biology, School of Life Sciences, Shandong University, Qingdao, China

[3] Current Address: School of Basic Medical Sciences, Shenzhen University Medical School, Shenzhen, China

[4] Howard Hughes Medical Institute, University of Texas Southwestern Medical Center, Dallas, TX 75390, USA

[*] Correspondence: sean.morrison@utsouthwestern.edu

This PDF file includes:

Supplementary figures and legends

Tables S1 to S3

Supplementary Materials and Methods

SI References

Supplemental figures and tables

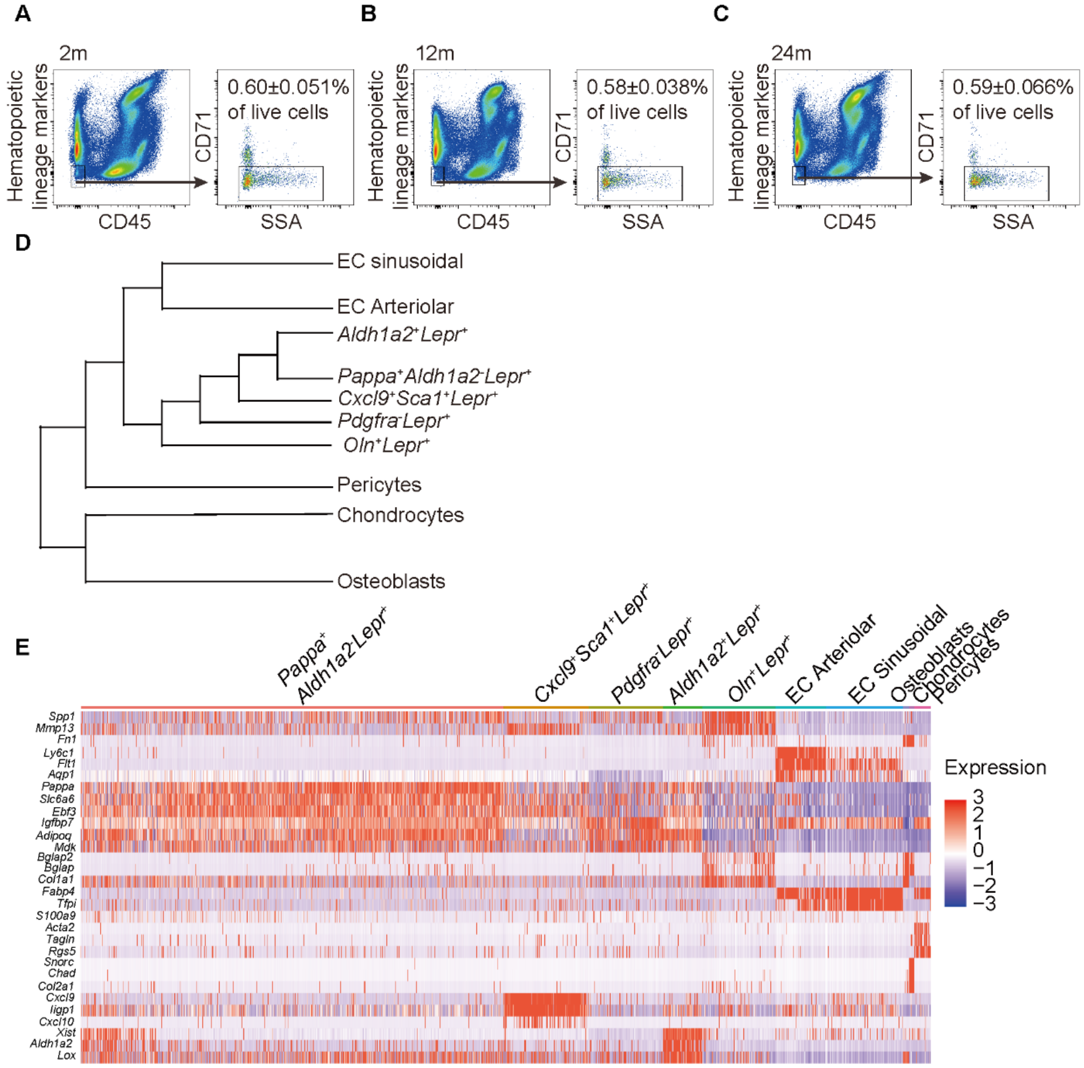

Fig. S1, Related to Figure 1. Single cell RNA sequencing identifies clusters of stromal cells in adult bone marrow.

(*A*-*C*) Representative flow cytometry gates used for the isolation of stromal cells that were negative for CD45, CD71, and hematopoietic lineage markers from enzymatically dissociated tibia and femur bone marrow of 2 (*A*), 12 (*B*), and 24 (*C*) month-old wild-type mice.

(*D*) Hierarchical clustering of stromal cell clusters from the single cell RNA sequencing data.

(*E*) Heatmap showing three genes per cell cluster that most distinguished each cell cluster from other cell clusters by single cell RNA sequencing.

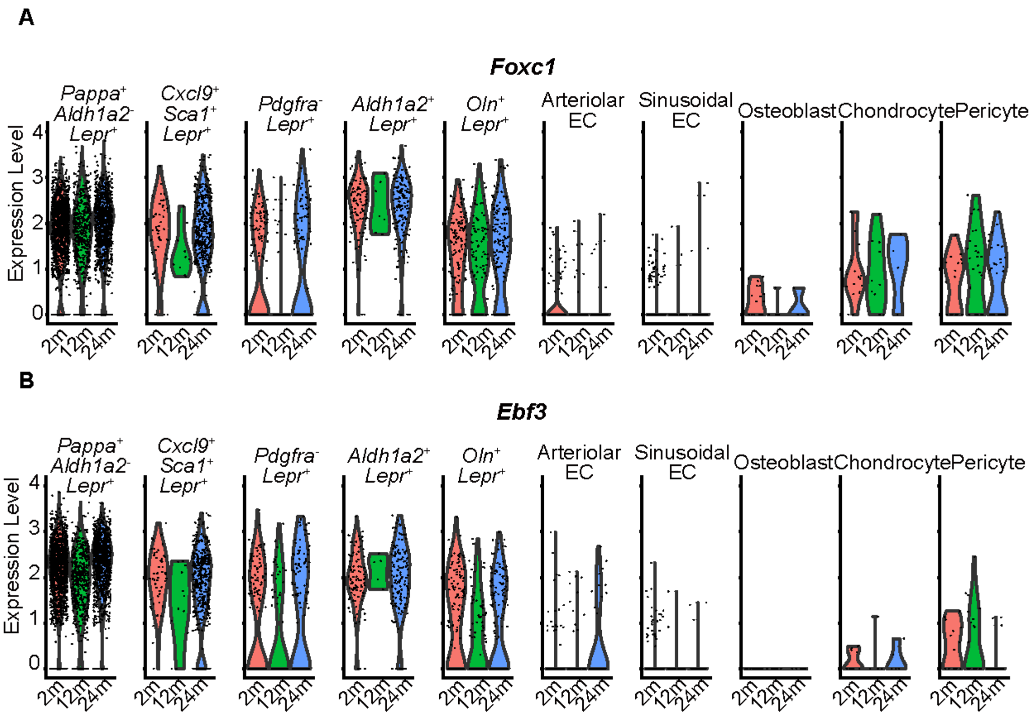

Fig. S2, Related to Figure 1. ***Foxc1*** **and** ***Ebf3*** **expression in bone marrow stromal cells from 2-, 12-, and 24-month-old mice.**

Violin plots showing *Foxc1* (*A*) and *Ebf3* (*B*) expression in each cell cluster at each age.

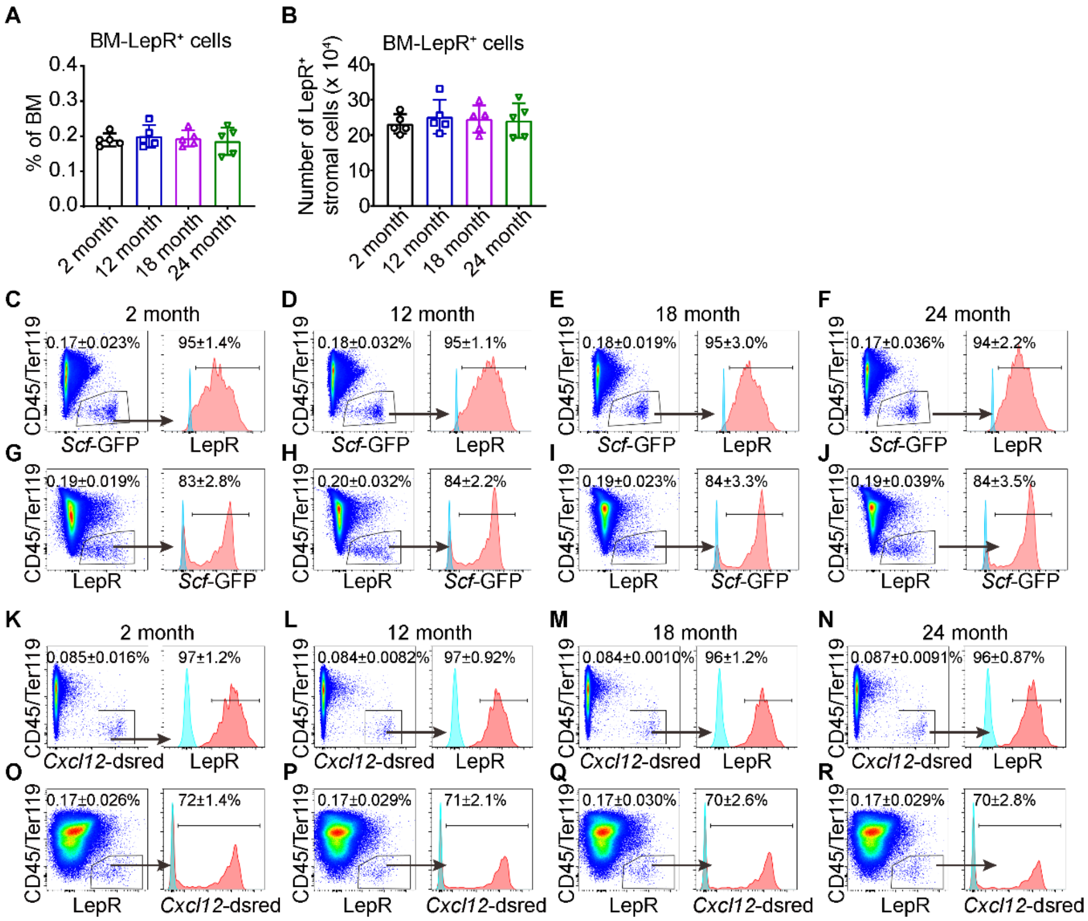

Fig. S3, Related to Figure 1. *Scf*-GFP and *Cxcl12*-DsRed expression in LepR+ stromal cells from the bone marrow of 2-, 12-, 18- and 24-month-old mice.

(*A, B*) The frequency (*A*) and absolute number *(B)* of LepR+ stromal cells in enzymatically dissociated bone marrow from mice at 2, 12, 18, and 24 months of age (a total of 5 mice per time point from 2 independent experiments per time point).

(*C*-*J*) Analysis of *Scf*-GFP and LepR staining in enzymatically dissociated bone marrow cells from *Scf*-GFP mice at 2 (*C* and *G*), 12 (*D* and *H*), 18 (*E* and *I*), and 24 (*F* and *J*) months of age. Panels *C-F* show the frequency of *Scf*-GFP+ stromal cells as a percentage of all bone marrow cells as well as the percentage of *Scf*-GFP+ stromal cells that were LepR+. Panels *G-J* show the frequency of LepR+ stromal cells as a percentage of all bone marrow cells as well as the percentage of LepR+ cells that were *Scf*-GFP+.

(*K-R*) Analysis of *Cxcl12*-DsRed and LepR staining in enzymatically dissociated bone marrow cells from *Cxcl12*-DsRed mice at 2 (*K* and *O*), 12 (*L* and *P*), 18 (*M* and *Q*), and 24 (*N* and *R*) months of age. Panels *K-N* show the frequency of *Cxcl12*-DsRed^{high} stromal cells as a percentage of all bone marrow cells as well as the percentage of *Cxcl12*-DsRed^{high} stromal cells that were LepR^{+}. Panels *O-R* show the frequency of LepR^{+} stromal cells as a percentage of all bone marrow cells as well as the percentage of LepR^{+} cells that were *Cxcl12*-DsRed^{high}.

All data represent mean ± standard deviation. Each dot in panel *A* and *B* represents a different mouse. All data in panels *C-R* are from a total of 5 mice per time point from 2 independent experiments per time point.

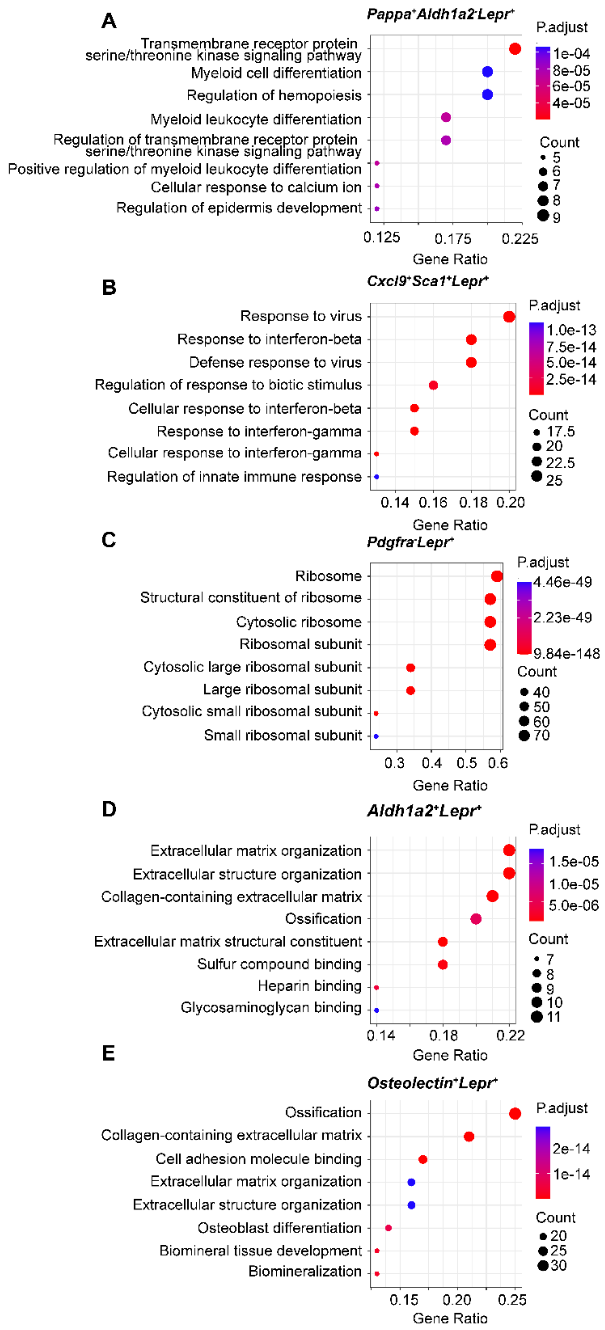

Fig. S4, Related to Figure 1. The top 8 most highly enriched gene ontology (GO) terms in each *Lepr*$^{+}$ cell cluster by single cell RNA sequencing. GO term enrichment analysis among genes that were differentially expressed among *Lepr*$^{+}$ cell clusters including *Pappa*$^{+}$*Aldh1a2*$^{-}$*Lepr*$^{+}$ cells (*A*), *Sca1*$^{+}$*Cxcl9*$^{+}$*Lepr*$^{+}$ cells (*B*), *Pdgfra*$^{-}$*Lepr*$^{+}$ cells (*C*), *Aldh1a2*$^{+}$*Lepr*$^{+}$ cells (*D*), and *Osteolectin*$^{+}$*Lepr*$^{+}$ cells (*E*).

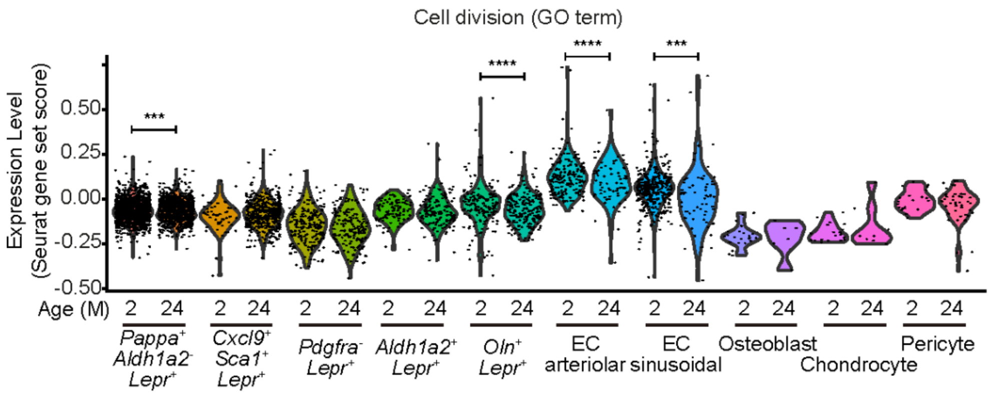

Fig. S5, Related to Figure 1 and Figure 5. Expression levels of cell cycle genes (From GO term "GOBP Cell Division") in bone marrow stromal cells at 2 and 24 months of age. In each case in which statistically significant differences were observed between 2 and 24 months of age, the average expression levels of cell cycle genes declined.

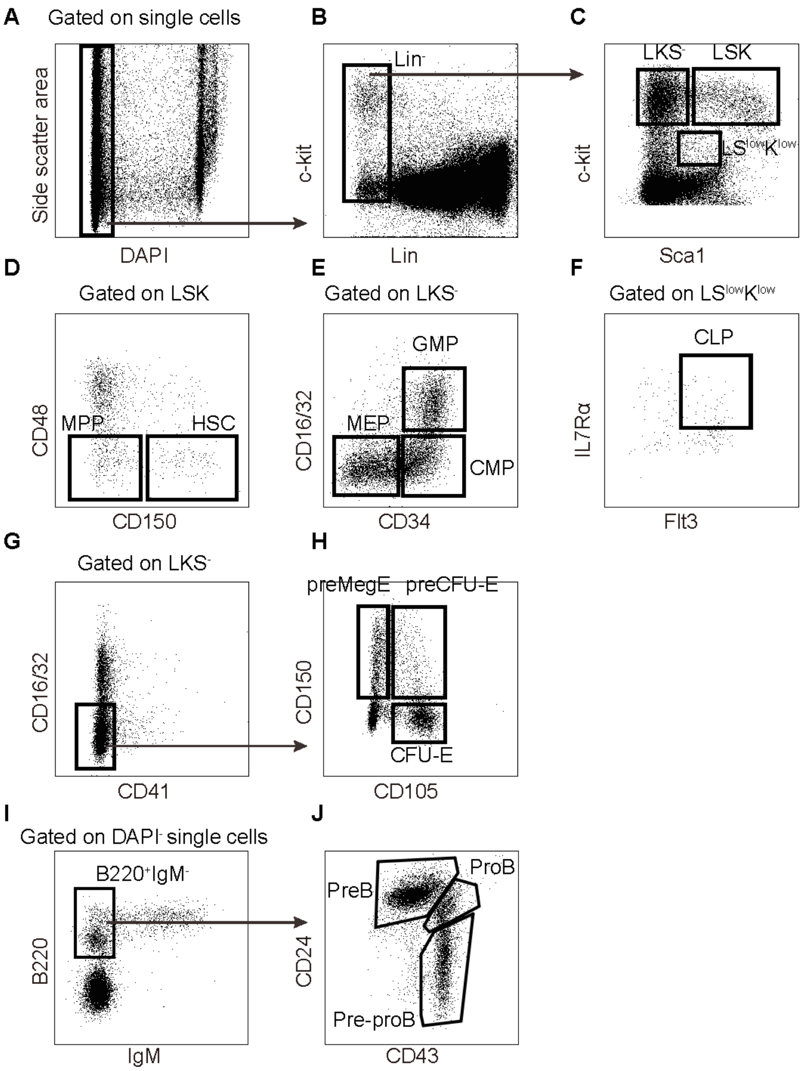

Fig. S6, Related to Figures 2, 4, and 6. Flow cytometry gating strategy for the identification of hematopoietic stem and progenitor cell populations. (*A-J*) Representative flow cytometry gates used to identify each of the hematopoietic stem and progenitor cell populations we characterized in the bone marrow.

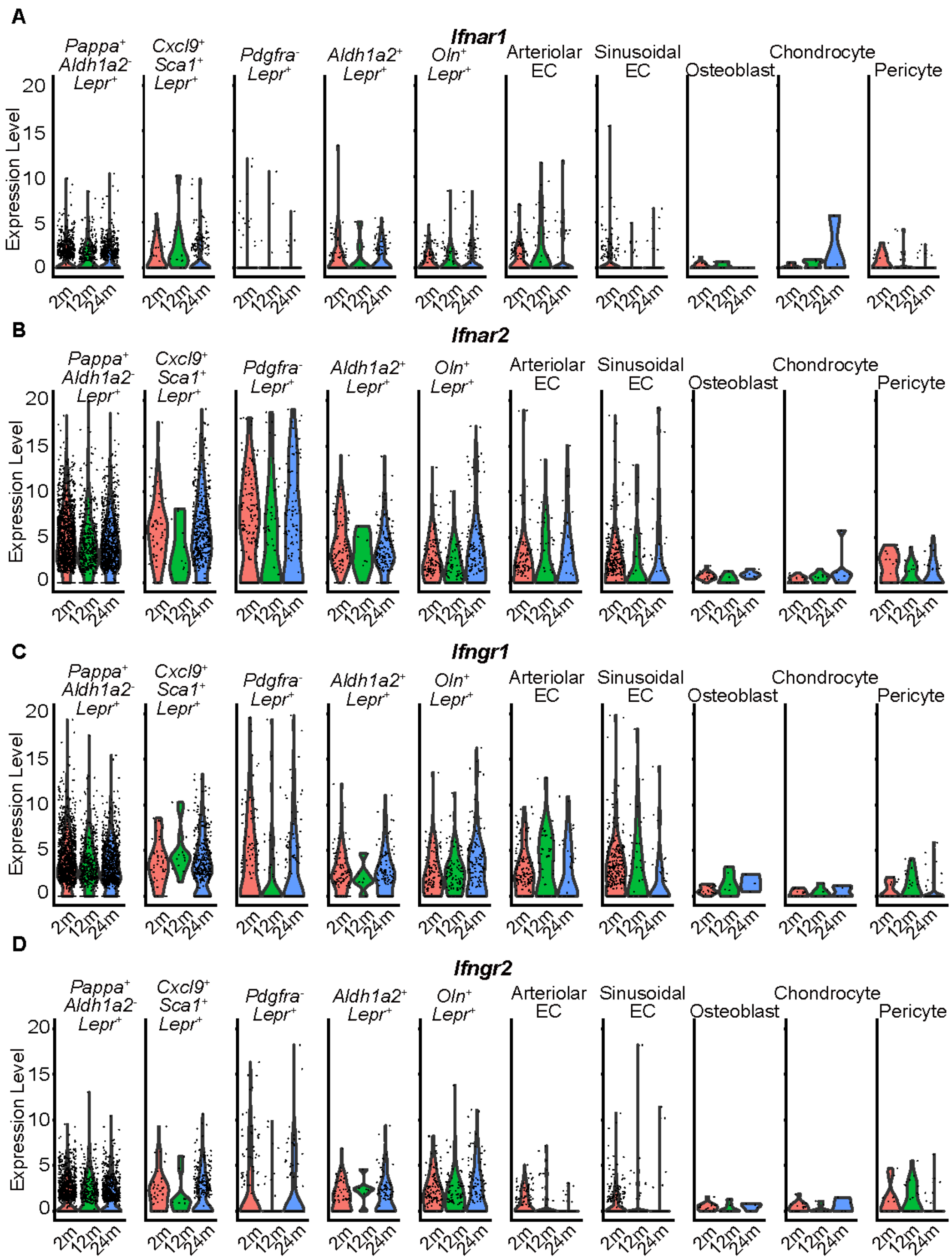

Fig. S7, Related to Figure 5. *Ifnar1*, *Ifnar2*, *Ifngr1* and *Ifngr2* expression by bone marrow stromal cells from 2-, 12-, and 24-month-old mice.

Violin plots showing *Ifnar1* (*A*), *Ifnar2* (*B*), *Ifngr1* (*C*), and *Ifngr2* (*D*) expression in each cell cluster at each age.

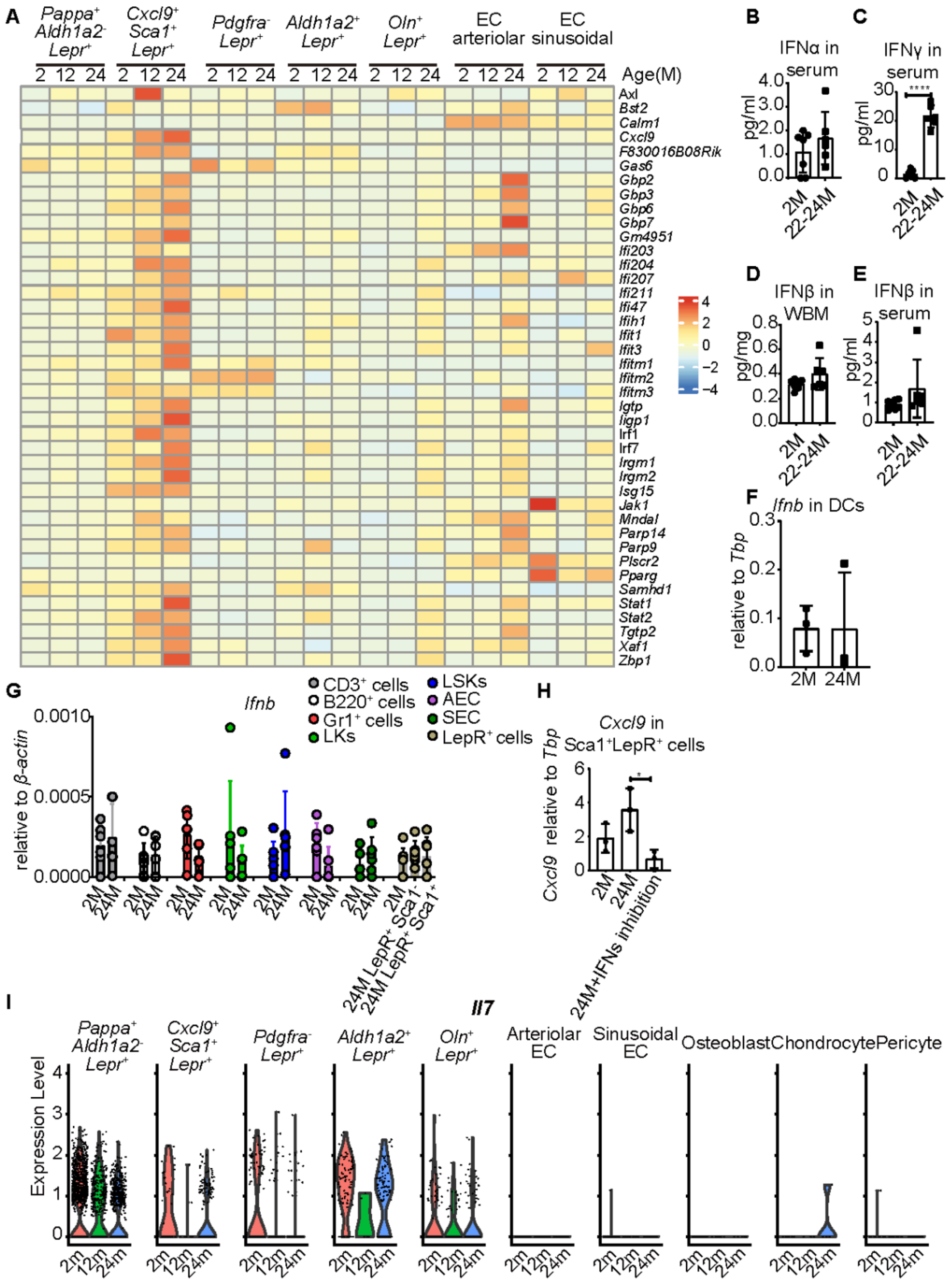

Fig. S8, Related to Figure 6. Interferon regulated genes increased in expression primarily within arteriolar endothelial cells and *Sca1*⁺*Cxcl9*⁺*Lepr*⁺ periarteriolar stromal cells in aging bone marrow.

(*A*) Heatmap showing the expression of interferon regulated genes in *Lepr*⁺ cell clusters,

arteriolar endothelial cells, and sinusoidal endothelial cells.

(*B* and *C*) The levels of IFNα (*B*) and IFNγ (*C*) by ELISA in blood serum (n=6-7 for each group).

(*D* and *E*) The levels of IFNβ by ELISA in bone marrow lysate (*D*) and blood serum (*E*), (n=6-7 for each group).

(*F*) *Ifnb* transcript levels by quantitative RT-PCR in dendritic cells from the bone marrow of 2- and 24-month-old mice (3 mice per time point).

(*G) Ifnb* transcript levels by quantitative RT-PCR in $CD3^+$ T cells, $B220^+$ B cells, $Gr1^+$ myeloid cells, LK myeloid progenitors, LSK stem/progenitor cells, arteriolar endothelial cells, sinusoidal endothelial cells, and $LepR^+$ stromal cells from the bone marrow of 2- and 24-month-old mice (6 mice per time point from 2 independent experiments).

(*H*) *Cxcl9* transcript levels by quantitative RT-PCR in $Sca1^+LepR^+$ stromal cells with or without treatment with anti-IFNAR and anti-IFNγ antibodies.

(*I*) Violin plots showing *Il7* expression in each cell cluster at each age.

Each dot represents a different mouse in panels *B-H*. All data represent mean ± standard deviation.

Table S1: related to Figure 1. The frequencies of each cell cluster as a percentage of bone marrow stromal cells at 2, 12, and 24 months of age based on single cell RNA sequencing.

Cluster number	Cluster name	% of stromal cells at 2M	% of stromal cells at 12M	% of stromal cells at 24M
1	*Pappa*$^{+}$*Aldh1a2*$^{-}$ *Lepr*$^{+}$	53	54	44
2	*Cxcl9*$^{+}$*Sca1*$^{+}$*Lepr*$^{+}$	2.3	1.4	22
3	*Pdgfra*$^{-}$*Lepr*$^{+}$	8.8	9.9	8.2
4	*Aldh1a2*$^{+}$*Lepr*$^{+}$	4.1	0.77	6.9
5	*Osteolectin*$^{+}$*Lepr*$^{+}$	6.7	12	9.4
6	Arteriolar endothelial cells	7.4	8.3	3.3
7	Sinusoidal endothelial cells	15	7.2	3.0
8	Osteoblasts	0.95	0.88	0.28
9	Chondrocytes	0.78	1.1	0.37
10	Pericytes	0.86	4.4	2.0

Table S2. Related to Figures 2, 3, 4, and 6. The markers used to identify each hematopoietic stem and progenitor cell population analyzed in this study.

Population	Abbreviation	Markers	References
Hematopoietic Stem Cells	HSC	$Lin^-c\text{-}kit^+Sca\text{-}1^+CD48^-CD150^+$	Ref(1, 2)
Multipotent Progenitors	MPP	$Lin^-c\text{-}kit^+Sca\text{-}1^+CD48^-CD150^-$	Ref(1, 2)
Megakaryocyte-Erythrocyte Progenitors	MEP	$Lin^-c\text{-}kit^+Sca\text{-}1^-CD16/32^-CD34^-$	Ref(2, 3)
Granulocyte-Macrophage Progenitors	GMP	$Lin^-c\text{-}kit^+Sca\text{-}1^-CD16/32^+CD34^+$	Ref(2, 3)
Common Myeloid Progenitors	CMP	$Lin^-c\text{-}kit^+Sca\text{-}1^-CD16/32^-CD34^+$	Ref(2, 3)
Common Lymphoid Progenitors	CLP	$Lin^-c\text{-}kit^{low}Sca\text{-}1^{low}IL7R\alpha^+Flt3^+$	Ref(2, 4)
Pre-Megakaryocyte-erythrocyte progenitors	PreMegE	$Lin^-c\text{-}kit^+Sca\text{-}1^-CD41^-CD16/32^-CD150^+CD105^-$	Ref(2, 5)
PreCFU-E progenitors	Pre-CFU-E	$Lin^-c\text{-}kit^+Sca\text{-}1^-CD41^-CD16/32^-CD150^+CD105^+$	Ref(2, 5)
CFU-E progenitors	CFU-E	$Lin^-c\text{-}kit^+Sca\text{-}1^-CD41^-CD16/32^-CD150^-CD105^+$	Ref(2, 5)
Pre-proB cells precursors	Pre-proB	$B220^+$ IgM^- $CD43^+$ $CD24^-$	Ref(6, 7)
Pro-B cells precursors	Pro-B	$B220^+$ IgM^- $CD43^+$ $CD24^+$	Ref(6, 7)
Pre-B cells precursors	Pre-B	$B220^+$ IgM^- $CD43^-$ $CD24^+$	Ref(6, 7)
B cells		$B220^+$	
T cells		$CD3^+$	
Granulocytes		$Mac1^+Gr1^+$	

Table S3. Related to Figure 3. Summary of the donor cell reconstitution profiles in primary and secondary recipient mice in Figure 3.

A

12M Primary transplantation								
**Donor mouse genotype**	**Recipient mice**	**Long-term**			**Transient**			**No reconstitution**
		**M+B+T**	**M+B**	**M**	**M+B+T**	**M+B**	**M**	
Scf$^{GFP/FL}$	14	100%	0%	0%	0%	0%	0%	0%
Tie2-cre;Scf$^{GFP/FL}$	15	100%	0%	0%	0%	0%	0%	0%
Lepr-cre;Scf$^{GFP/FL}$	15	100%	0%	0%	0%	0%	0%	0%
Lepr-cre;Tie2-cre; Scf$^{GFP/FL}$	14	93%	7%	0%	0%	0%	0%	0%

B

12M Secondary transplantation								
**Donor mouse genotype**	**Recipient mice**	**Long-term**			**Transient**			**No reconstitution**
		**M+B+T**	**M+B**	**M**	**M+B+T**	**M+B**	**M**	
Scf$^{GFP/FL}$	10	100%	0%	0%	0%	0%	0%	0%
Tie2-cre;Scf$^{GFP/FL}$	10	100%	0%	0%	0%	0%	0%	0%
Lepr-cre;Scf$^{GFP/FL}$	10	100%	0%	0%	0%	0%	0%	0%
Lepr-cre;Tie2-cre; Scf$^{GFP/FL}$	10	0%	0%	100%	0%	0%	0%	0%

C

18M Primary transplantation								
**Donor mouse genotype**	**Recipient mice**	**Long-term**			**Transient**			**No reconstitution**
		**M+B+T**	**M+B**	**M**	**M+B+T**	**M+B**	**M**	
Scf$^{GFP/FL}$	14	100%	0%	0%	0%	0%	0%	0%
Tie2-cre;Scf$^{GFP/FL}$	14	100%	0%	0%	0%	0%	0%	0%
Lepr-cre;Scf$^{GFP/FL}$	15	100%	0%	0%	0%	0%	0%	0%
Lepr-cre;Tie2-cre; Scf$^{GFP/FL}$	14	100%	0%	0%	0%	0%	0%	0%

D

18M Secondary transplantation								
**Donor mouse genotype**	**Recipient mice**	**Long-term**			**Transient**			**No reconstitution**
		**M+B+T**	**M+B**	**M**	**M+B+T**	**M+B**	**M**	
Scf$^{GFP/FL}$	10	100%	0%	0%	0%	0%	0%	0%
Tie2-cre;Scf$^{GFP/FL}$	10	100%	0%	0%	0%	0%	0%	0%
Lepr-cre;Scf$^{GFP/FL}$	10	90%	10%	0%	0%	0%	0%	0%
Lepr-cre;Tie2-cre; Scf$^{GFP/FL}$	10	30%	30%	0%	10%	0%	10%	20%

E

24M Primary transplantation								
**Donor mouse genotype**	**Recipient mice**	**Long-term**			**Transient**			**No reconstitution**
		**M+B+T**	**M+B**	**M**	**M+B+T**	**M+B**	**M**	
Scf$^{GFP/FL}$	15	100%	0%	0%	0%	0%	0%	0%
Tie2-cre;Scf$^{GFP/FL}$	14	100%	0%	0%	0%	0%	0%	0%
Lepr-cre;Scf$^{GFP/FL}$	14	86%	7%	0%	7%	0%	0%	0%
Lepr-cre;Tie2-cre; Scf$^{GFP/FL}$	14	50%	0%	0%	43%	7%	0%	0%

F

24M Secondary transplantation								
**Donor mouse genotype**	**Recipient mice**	**Long-term**			**Transient**			**No reconstitution**
		**M+B+T**	**M+B**	**M**	**M+B+T**	**M+B**	**M**	
Scf$^{GFP/FL}$	10	100%	0%	0%	0%	0%	0%	0%
Tie2-cre; Scf$^{GFP/FL}$	10	100%	0%	0%	0%	0%	0%	0%
Lepr-cre; Scf$^{GFP/FL}$	10	30%	10%	0%	10%	10%	40%	0%
Lepr-cre; Tie2-cre; Scf$^{GFP/FL}$	10	10%	0%	10%	30%	0%	10%	40%

Supplementary Materials and Methods

Single cell RNA sequencing

Whole tibias and femurs were dissociated as described previously(8). Cells were stained with antibodies against CD45, CD71, CD2, CD3, CD5, CD8, CD11b, Ter-119, B220 and Gr-1. Dead cells and debris were excluded by gating on forward scatter, side scatter, and the viability dye 4′,6-diamidino-2-phenylindole (DAPI; 2 μg/ml). Bone marrow stromal cells were isolated by sorting live cells that were negative for hematopoietic markers. 15,000 of these cells were sorted into staining medium, then pelleted by centrifugation and resuspended in 40 μl of staining medium. Single cell RNA sequencing libraries were generated using Chromium Next GEM Single Cell 3' Reagent Kits v3.1 (10xGenomics) according to the manufacturer's instructions. Briefly, cells, reagents (10xGenomics, PN-1000130), gel beads (10xGenomics, 2000164), and partitioning oil (10xGenomics, 220088) were loaded into the Chromium Next GEM Chip G (10xGenomics, 200177). The chip was loaded into the Chromium Controller (10xGenomics) to generate Gel Bead-In-Emulsions (GEMs). After reverse transcription using the GEM-RT incubation protocol, cDNA was extracted from GEMs using the recovery reagent (10xGenomics, 220016), cDNA was purified with DynaBeads MyOne Silane beads (10xGenomics, 2000048), amplified by PCR for 12 cycles, and further purified with SPRIselect reagent (Beckman Coulter, B23318). The DNA concentration and amplicon size were measured using a Tapestation (Agilent) with D1000 high sensitivity screentapes (Agilent, 5067-5582), and cDNA yield was measured using the Qubit DNA high sensitivity assay (Invitrogen, Q32854), to ensure the amplified cDNA met quality control standards. After fragmentation, end-repair, A-tailing and size selection, cDNA was ligated with adaptor and purified with SPRIselect reagents, according to 10xGenomics instructions. Libraries were amplified by PCR with sample index primers from the Chromium i7 Multiplex kit (10xGenomics, PN-120262), typically for a total of 14 cycles, depending on the original cDNA yield measured by Qubit. Final PCR products were subjected to double-sided size selection with SPRIselect reagents to eliminate fragments larger

and smaller than the target amplicons. Library cDNA concentration and size were determined using the Agilent Tapestation with D1000 screentapes (Agilent, 5067-5582), as well as the Qubit DNA high sensitivity assay. cDNA libraries were sequenced using a NextSeq 500 sequencer using the 150bp high output sequencing kit (Illumina), with the following pair-end sequencing settings: Read 1 - 28bp, i7 index – 10bp, i5 index – 10bp, Read 2 - 90bp, generating ~400 million raw reads per run(8).

Primary cell culture

To perform colony forming assays, $1x10^4$ bone marrow cells were plated in 60mm petri dishes (Falcon) containing 5ml Methocult M3434 medium (StemCell Technologies) supplemented with 10ng/ml of recombinant thrombopoietin (Peprotech) and penicillin/streptomycin (Fisher Scientific). After 7 days of culture at 37°C, colonies were counted using an inverted microscope.

Measurement of interferons by ELISA

Serum was obtained from the blood after centrifugation at 900 x g for 10 minutes to eliminate cells. Bone marrow was lysed in protein lysis buffer (RIPA buffer with protease and phosphatase inhibitors). Interferons were measured using PBL high-sensitivity (IFNα and IFNβ) mouse ELISA kits (PBL Assay), and Legend MAX™ Mouse ELISA kits (IFNγ) (BioLegend) according to the manufacturer's instructions.

Bone sectioning, immunostaining and confocal imaging

Freshly dissected mouse femurs were fixed in 4% paraformaldehyde (Fisher Scientific) overnight. Bones were decalcified in PBS with 0.5M EDTA for 14 days, followed by cryopreservation in 30% sucrose for one day. Bones were sectioned into 30μm slices using the CryoJane system (Leica). Sections were blocked in PBS with 5% normal donkey serum

(Jackson ImmunoResearch) for 1 hour and then stained overnight with goat-anti-LepR (1:50, R&D Systems), rat-anti-Sca1 (1:100, BioLegend), rabbit-anti-laminin (1:200, Abcam), and/or anti-CD3-APC (Tonbo Biosciences, 1:100) antibodies. Donkey-anti-rabbit Alexa Fluor 488 (1:200, Jackson ImmunoResearch), donkey-anti-goat Alexa Fluor 555 (1:500, Life Technologies), Donkey-anti-rat Alexa Fluor 488 (1:200, Fisher Scientific), and/or Donkey-anti rabbit Alexa Fluor 647 (1:200, Jackson ImmunoResearch) were used as secondary antibodies. Slides were mounted with anti-fade prolong gold (Life Technologies). Images were acquired with a Zeiss LSM780 confocal microscope and quantified using Bitplane Imaris V9.0(2, 9).

We used Bitplane Imaris V10.1 to calculate the distance from $CD3^+$ cells or $Sca1^+LepR^+$ cells to arterioles and sinusoids in thick femur sections as previously reported (2). We used the Imaris Spot function to identify and label $CD3^+$ cells and $Sca1^+LepR^+$ cells as digital spots, and the Imaris Surface function to create a digital surface for blood vessels based on anti-laminin staining. To distinguish between arterioles and sinusoids, the anti-laminin channel was first segmented manually based on blood vessel morphology and then masked to produce two channels, one representing arterioles and the other representing sinusoids. Three-dimensional distances from $CD3^+$ cells or $Sca1^+LepR^+$ cells and these digital surfaces were calculated using spot to surface statistics in Andor Imaris image analysis software. The distance from random spots to arterioles and sinusoids was automatically calculated using the Imaris Vantage module(2).

Single cell RNA sequencing data analysis

Cell Ranger single cell software suite (version 6.1.2, https://support.10xgenomics.com/single-cell-gene-expression/software/overview/welcome) was used to perform sample demultiplexing, alignment, filtering, and UMI counting. In total, we analyzed 9,240 cells from 2-month-old mice, with 48,108 reads per cell and 74.6% of the reads were uniquely mapped to Cell Ranger's mouse reference genome mm10-2020-A. The median

UMIs and genes per cell for the 2-month-old mice were 6,562 and 1,802, respectively. We sequenced 9,079 cells from 12-month-old mice, with 51,474 reads per cell and 83.9% of the reads were uniquely mapped to the mouse reference genome. The median UMIs and genes per cell for the 12-month-old mice were 5,752 and 1,490, respectively. We sequenced 12,420 cells, with 37,829 reads per cell from 24-month-old mice and 79.9% of reads uniquely mapped to the mouse reference genome. The median UMIs and genes per cell for the 24-month-old mice were 4,964 and 1,512, respectively. The numbers and distribution of detected genes per cell were comparable across samples.

Cell quality filtering, normalization, sample integration, clustering, gene expression, and data visualization were analyzed with the Seurat package (version 4.2.3) (10) on R (version 4.0.2). Cells were first filtered to have 200 to 10,000 detected genes, 500 to 100,000 UMI counts, and less than 7.5% of total UMIs mapping to the mitochondrial genome. After quality filtering and removing unwanted cells from the data, we applied Seurat's SCTransform on the UMI counts to account for the variability among cells caused by different sequencing depths and potential batch effects. We avoided clustering cells based on differences in cell cycle status or transcription of mitochondrial genes by subtracting such differences from the data used for clustering. The 3,000 most variable genes across the transformed cells were selected using SelectIntegrationFeatures, and samples were integrated using those genes with an anchor set of cells found by FindIntegrationAnchors. Integrated data were reduced to 30 dimensions by RunPCA. Dimension-reduced cells were clustered by FindNeighbors followed by FindClusters, and UMAP plots were generated by RunUMAP using the integrated, dimension-reduced cells. For the gene expression analyses, we normalized the UMI counts by the total UMIs per cell, multiplied by a scale factor of 10,000 and log-transformed (log1p) the result. Normalized data were used for gene-expression visualizations. Heatmaps were based on the z-scores of normalized and log-transformed UMI counts.

We identified 27 clusters, with 17 of them expressing hematopoietic cell markers,

representing contaminating hematopoietic cells. These hematopoietic clusters were removed, and the remaining 10 clusters of non-hematopoietic stromal cells were analyzed further.

Analysis of differential gene expression among cell clusters was performed using the FindAllMarkers function with the Wilcoxon rank sum test. Markers that were used to identify and discriminate cell clusters were selected for having Bonferroni-adjusted $p<0.05$ for pairwise comparisons among clusters, expression by >10% cells in a cluster, and fold change >1.5 between clusters. Violin plots, heatmaps, the UMAP plot, and the clustering dendrogram were generated using Seurat's VlnPlot, DoHeatmap, FeaturePlot, and PlotClusterTree functions, respectively. Spearman correlation coefficients were calculated between samples from 2-month and 24-month-old mice for each cell cluster using average gene expression values.

A list of genes involved in the interferon signaling pathway was curated using Gene Ontology (GO) terms: GOBP response to interferon alpha, GOBP response to interferon beta, GOBP interferon mediated signaling pathway, and GOBP type II interferon mediated signaling pathway. Seurat's AddModuleScore function was used to calculate a per-cell score and a heatmap of interferon regulated genes.

RNA extraction and real-time qRT-PCR

For RNA extraction from sorted cells, 5,000–15,000 cells were sorted into 300 μl of buffer RLT (Qiagen RNAeasy Micro kit) and RNA was purified according to the manufacturer's instructions. RNA was reverse transcribed using iScript Reverse Transcription Supermix (Bio-Rad). The primers used for quantitative RT-PCR (qRT-PCR) analysis included *Ifna*: 5′-GGACTTTGGATTCCCGCAGGAGAAG-3′ and 5′- GCTGCATCAGACAGCCTTGCAGGTC-3′ (ref)(11); *Ifnb*: 5′- TCCGAGCAGAGATCTTCAGGAA-3′ and 5′-TGCAACCACCACTCATTCTGAG-3′ (ref)(11); *Ifng*: 5′-TTTGCAGCTCTTCCTCATGGCTGTTTCTG-3′ and 5′-TGACGCTTATGTTGTTGCTGATGGCCTG-3′ (ref)(11); and *Actb*: 5′-

GGCTGTATTCCCCTCCATCG-3′ and 5′- CCAGTTGGTAACAATGCCATGT-3′ (ref)(12); *Tbp*: 5′- AGAACAATCCAGACTAGCAGCA-3′ and 5′- GGGAACTTCACATCACAGCTC-3′; *Cxcl9*: 5′- GGAGTTCGAGGAACCCTAGTG-3′ and 5′- GGGATTTGTAGTGGATCGTGC-3′; *Cfb*: 5′- GAGCGCAACTCCAGTGCTT-3′ and 5′- GAGGGACATAGGTACTCCAGG-3′. Transcript levels were normalized to *Actb* or *Tbp* (TATA box binding protein) and fold change was calculated using the ΔCt method.

Bone marrow transplantation assays

Recipient mice (CD45.1/CD45.2) were irradiated using an XRAD 320 X-ray irradiator (Precision X-Ray Inc.) with two dose of 540 rad at least 4 hours apart. 500,000 unfractionated bone marrow cells from each of donor (CD45.2) and competitor (CD45.1) mice were mixed and injected intravenously through the tail vein. For secondary bone marrow transplantation, 5 million unfractionated bone marrow cells from primary recipient mice were injected intravenously non-competitively into irradiated recipient mice through the tail vein. Recipient mice were bled every 4 weeks until 16 weeks to examine the frequency of donor-derived $CD45^+$, myeloid, B, and T cells. The blood was collected into EDTA KE/1.3 (Fisher Scientific) tubes to prevent clotting, then subjected to ammonium-chloride potassium chloride red cell lysis. Cells were then stained with fluorochrome-conjugated antibodies against CD45.1, CD45.2, Mac-1, B220, Gr1, and CD3. The samples were then analyzed using FACSAsria II (BD Bioscience) or FACSAria Fusion (BD Bioscience) flow cytometers(2, 9).

Statistical methods

Mice were allocated to experiments randomly and samples processed in an arbitrary order, but formal randomization techniques were not used. No formal blinding was applied when performing the experiments or analyzing the data. Samples sizes were not pre-determined based on statistical power calculations but were based on our experience with these assays. No

data were excluded.

Prior to analyzing the statistical significance of differences among groups, we tested whether data were normally distributed and whether variance was similar among groups. To test for normality, we performed the Shapiro–Wilk tests when $3 \leq n < 20$ or D'Agostino Omnibus tests when $n \geq 20$. To test whether variability significantly differed among groups we performed F-tests (for experiments with two groups) or Levene's median tests (for experiments with more than two groups). When the data significantly deviated from normality or variability significantly differed among groups, we log2-transformed the data and tested again for normality and variability. If the transformed data no longer significantly deviated from normality and equal variability, we performed parametric tests on the transformed data. If log2-transformation was not possible or the transformed data still significantly deviated from normality or equal variability, we performed non-parametric tests on the non-transformed data.

When data or log2-transformed data were normal and equally variable, statistical analyses were performed using Student's t-tests (when there were two groups), one-way ANOVAs (when there were more than two groups), two-way ANOVAs (when there were unpaired groups measured at different ages), or matched samples two-way ANOVAs (when there were matched groups measured at different ages). When the data or log2-transformed data were normal but unequally variable, statistical analyses were performed using Welch's t-tests (two groups) or Welch's one-way ANOVAs (more than two groups). When the data and log2-transformed data were abnormal or unequally variable, statistical analysis was performed using Mann-Whitney (when there were two groups) or Kruskal-Wallis tests (when there were more than two groups). P-values from multiple comparisons were adjusted using Holm-Sidak's method after Student's t-tests or Mann-Whitney tests, Tukey's method after one-way or two-way ANOVAs (when all the pairwise comparisons were interested), Sidak's method after matched samples two-way ANOVAs, Dunnett's method after one-way ANOVAs (when only the comparisons between a control and other groups were interested), Dunn's method after

Kruskal-Wallis tests, or Dunnett's T3 method after Welch's one-way ANOVAs. All statistical tests were two-sided. All data represent mean ± standard deviation. Statistical tests were performed using GraphPad Prism (10.1.2) or R (4.0.2).

References

1. O. H. Yilmaz, M. J. Kiel, S. J. Morrison, SLAM family markers are conserved among hematopoietic stem cells from old and reconstituted mice and markedly increase their purity. *Blood* **107**, 924-930 (2006).
2. S. Comazzetto *et al.*, Restricted Hematopoietic Progenitors and Erythropoiesis Require SCF from Leptin Receptor+ Niche Cells in the Bone Marrow. *Cell Stem Cell* **24**, 477-486 e476 (2019).
3. K. Akashi, D. Traver, T. Miyamoto, I. L. Weissman, A clonogenic common myeloid progenitor that gives rise to all myeloid lineages. *Nature* **404**, 193-197 (2000).
4. M. Kondo, I. L. Weissman, K. Akashi, Identification of clonogenic common lymphoid progenitors in mouse bone marrow. *Cell* **91**, 661-672 (1997).
5. C. J. Pronk *et al.*, Elucidation of the phenotypic, functional, and molecular topography of a myeloerythroid progenitor cell hierarchy. *Cell Stem Cell* **1**, 428-442 (2007).
6. R. R. Hardy, C. E. Carmack, S. A. Shinton, J. D. Kemp, K. Hayakawa, Resolution and characterization of pro-B and pre-pro-B cell stages in normal mouse bone marrow. *J Exp Med* **173**, 1213-1225 (1991).
7. B. Shen *et al.*, A mechanosensitive peri-arteriolar niche for osteogenesis and lymphopoiesis. *Nature* **591**, 438-444 (2021).
8. N. Kara *et al.*, Endothelial and Leptin Receptor(+) cells promote the maintenance of stem cells and hematopoiesis in early postnatal murine bone marrow. *Dev Cell* **58**, 348-360 e346 (2023).
9. B. O. Zhou *et al.*, Bone marrow adipocytes promote the regeneration of stem cells and haematopoiesis by secreting SCF. *Nat Cell Biol* **19**, 891-903 (2017).
10. T. Stuart *et al.*, Comprehensive Integration of Single-Cell Data. *Cell* **177**, 1888-1902 e1821 (2019).
11. S. Yum, M. Li, Y. Fang, Z. J. Chen, TBK1 recruitment to STING activates both IRF3 and NF-kappaB that mediate immune defense against tumors and viral infections. *Proc Natl Acad Sci U S A* **118** (2021).
12. P. Y. Huang, J. H. Guo, L. H. Hwang, Oncolytic Sindbis virus targets tumors defective in the interferon response and induces significant bystander antitumor immunity in vivo. *Mol Ther* **20**, 298-305 (2012).
